# Supplementary material for: Spatial coherence in DNA barcode networks
Source: Patterns (N Y). 2025 Dec 1;6(12):101428. doi: 10.1016/j.patter.2025.101428 (PMC12745985; doi:10.1016/j.patter.2025.101428)
Supplement: Document S1. Figures S1–S12 [file mmc1.pdf]

Patterns, Volume *6*

Supplemental information

Spatial coherence in DNA barcode networks

David Fernandez Bonet, Johanna I. Blumenthal, Shuai Lang, Simon K. Dahlberg, and Ian T. Hoffecker

Supplemental Figures

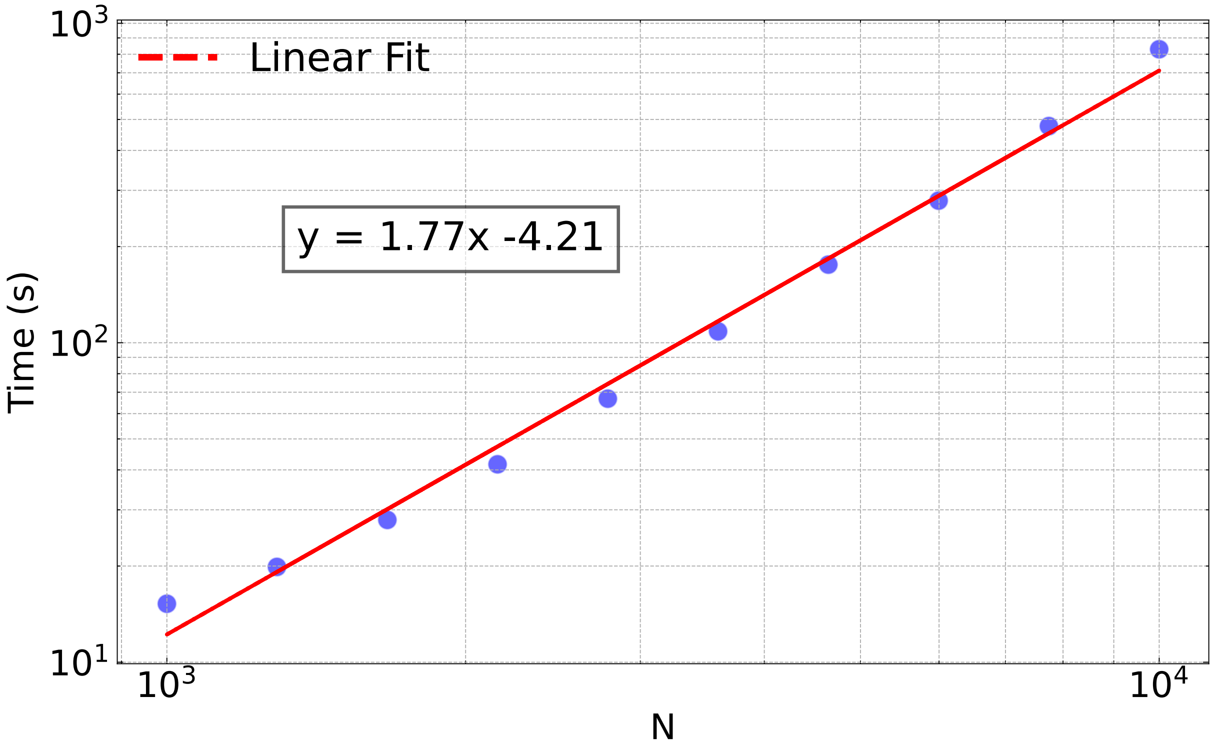

Figure S1: Empirical analysis of the time complexity associated with the spatial coherence pipeline. Computational time scales with varying input graph sizes, with a sub-quadratic scaling of 1.77.

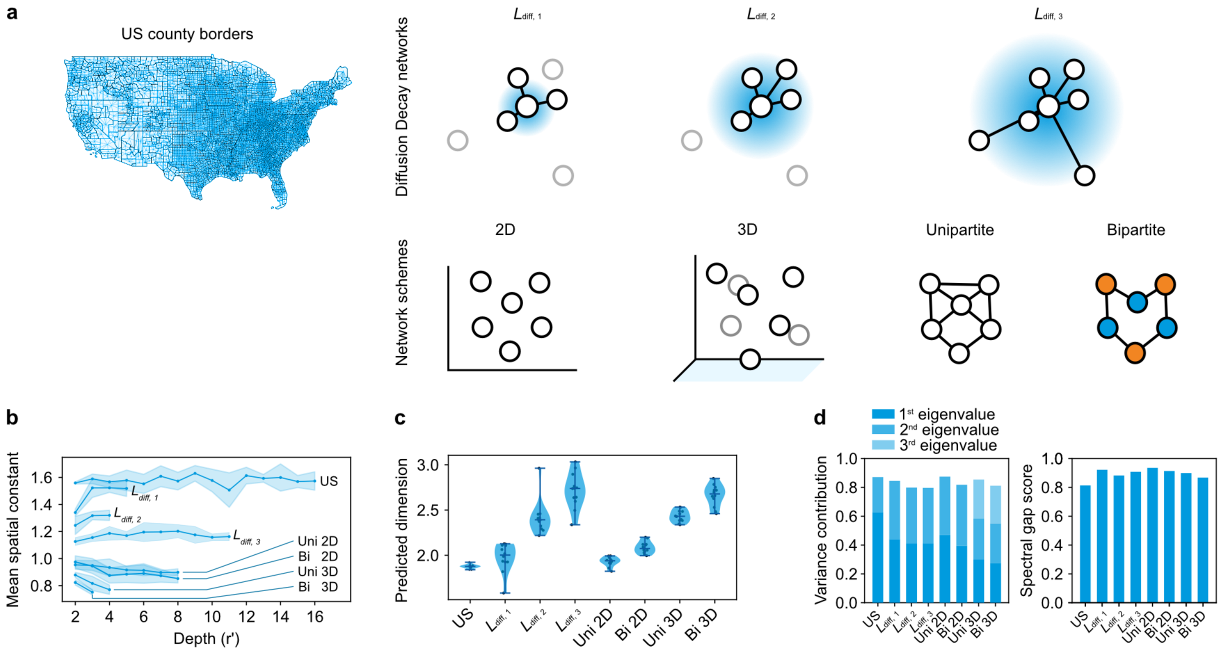

Figure S2: Spatial coherence is detectable in the topology of spatial networks (a) Different network modalities, including 2D/3D networks, unipartite/bipartite networks, and a distance decay based on diffusion to model interaction probabilities. Additionally, the US county borders network is included as a case study. (b) Spatial constant against depth revealing stable profiles for the spatial networks examined. (c) Network dimension values and (d) spectral analysis of the Gram matrix: variance contribution and spectral gap score showing values for spatial networks approaching 1.

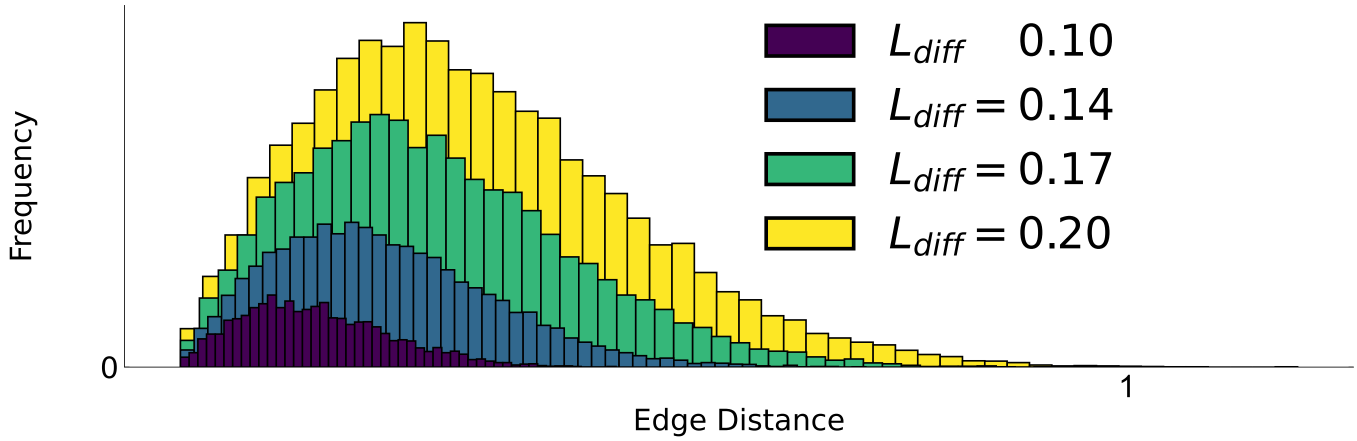

Figure S3: Edge length distribution for different characteristic diffusion lengths L_{diff}, used for network diffusion modeling.

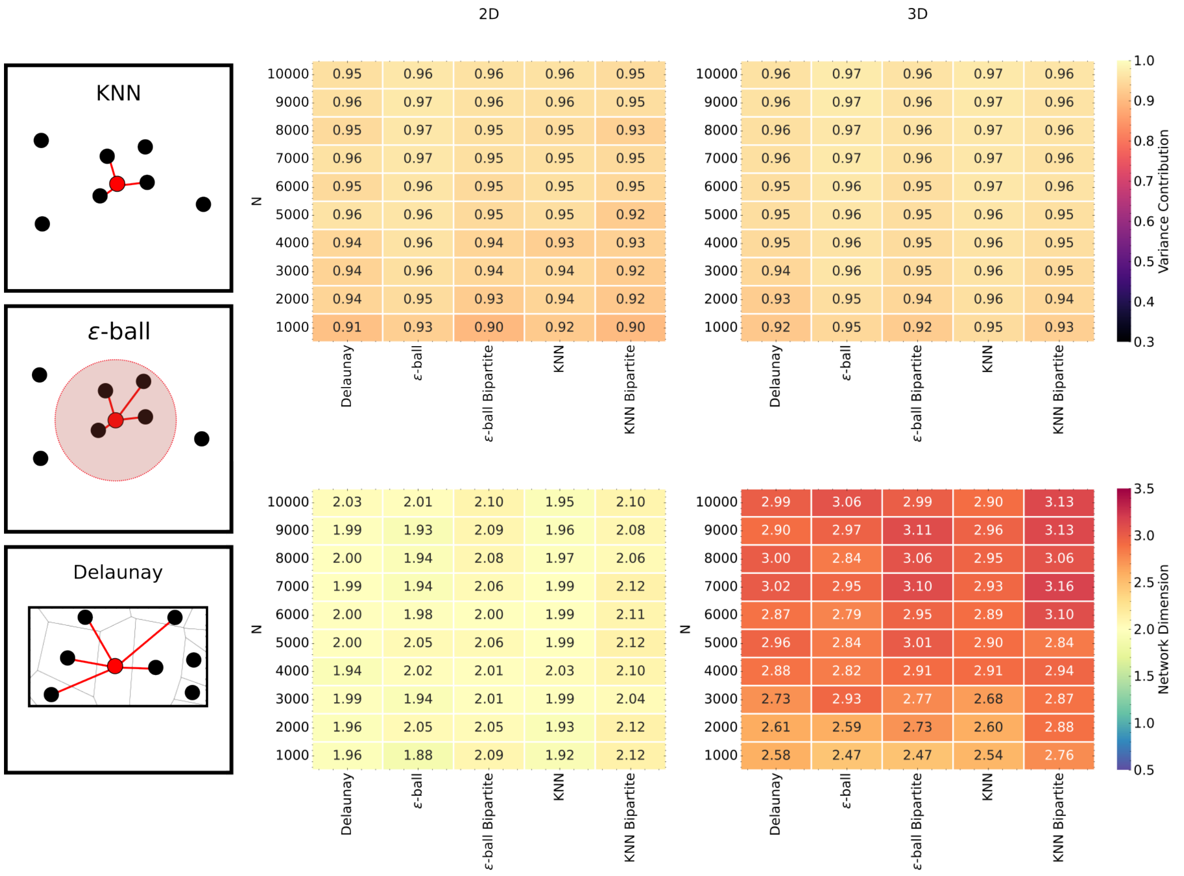

Figure S4: Spatial coherence measures analyzed across different numbers of nodes and proximity graph modalities in both 2D and 3D configurations, showing consistent results despite topological differences.

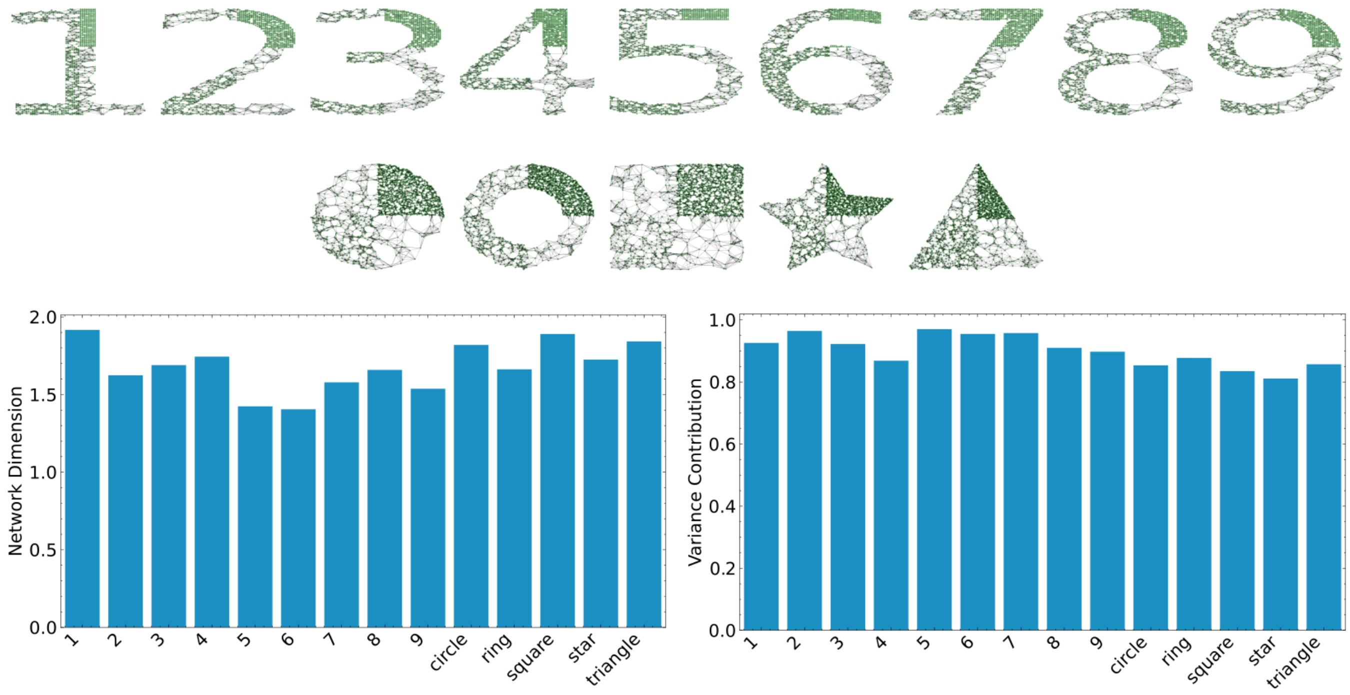

Figure S5: Spatial coherence measures on point clouds with different shapes, with variation regarding network dimension for shapes that are closer to a one-dimensional line and consistency for the variance contribution of the Gram matrix.

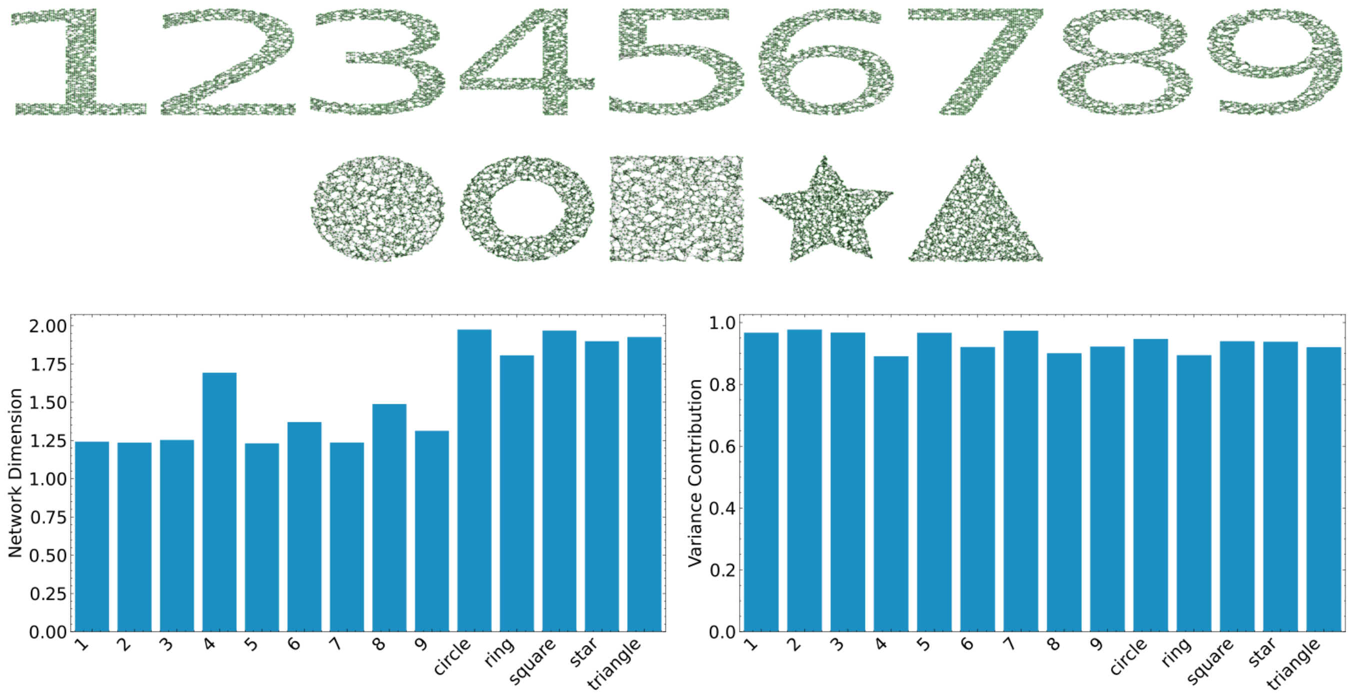

Figure S6: Spatial coherence measures on point clouds with different shapes and varying density regions, showing consistent values for spatial coherence measurements.

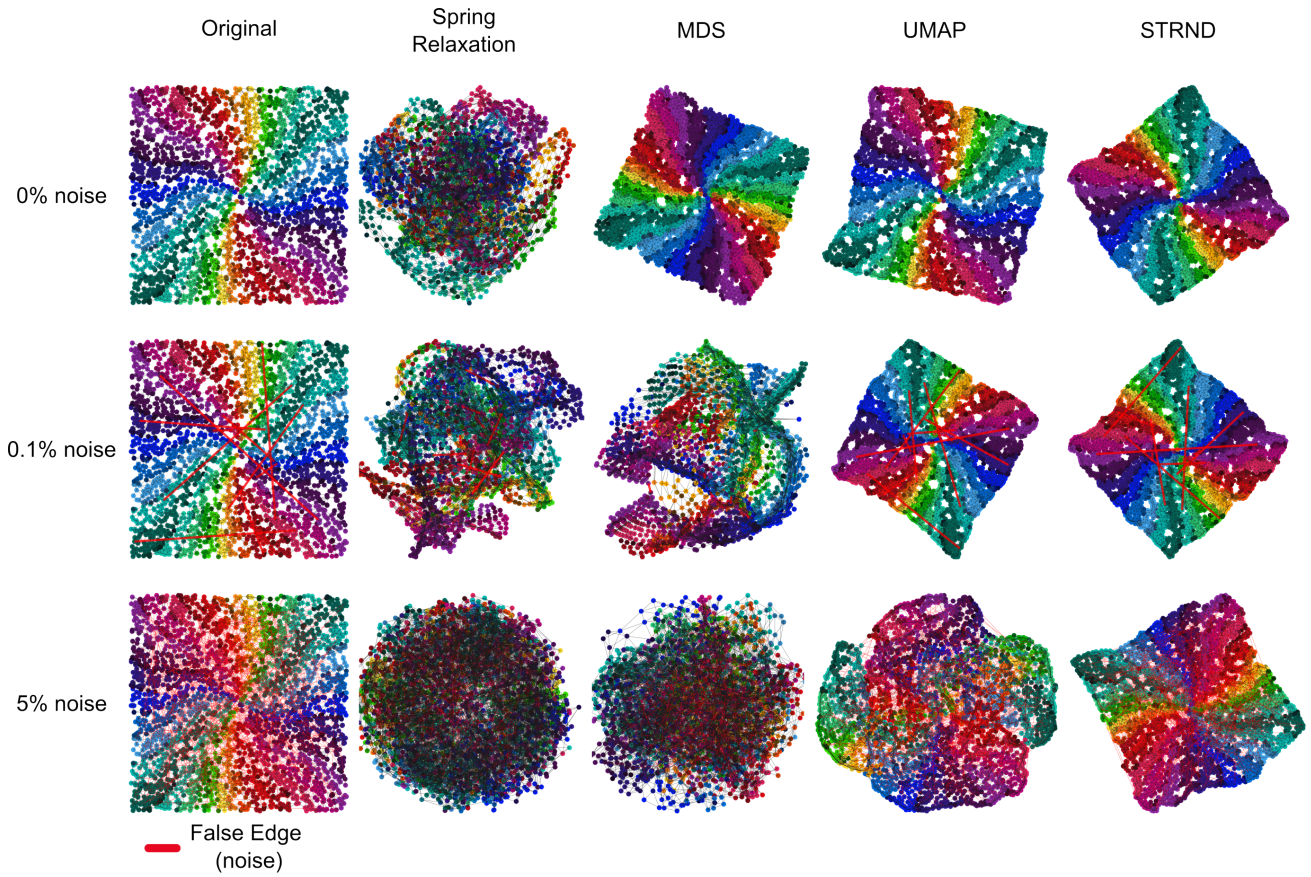

Figure S7: Image reconstruction visualizations for increasing noise levels (0%, 0.1%, 5%) on different image reconstruction methods.

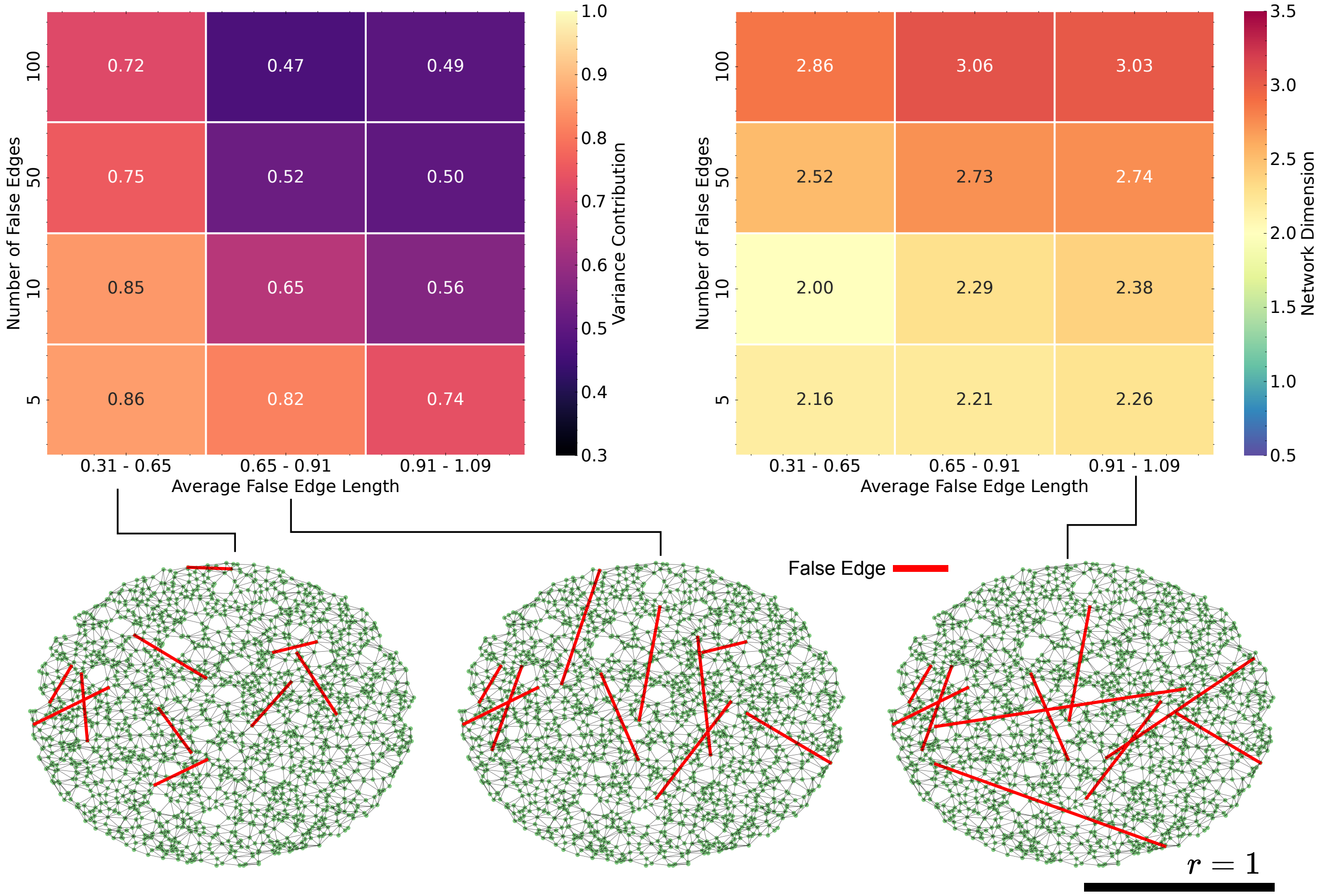

Figure S8: Impact of the number and length of false edges on spatial coherence measures. Left: variance contribution. Right: network dimension. A false edge length increase leads to a decrease in spatial coherence.

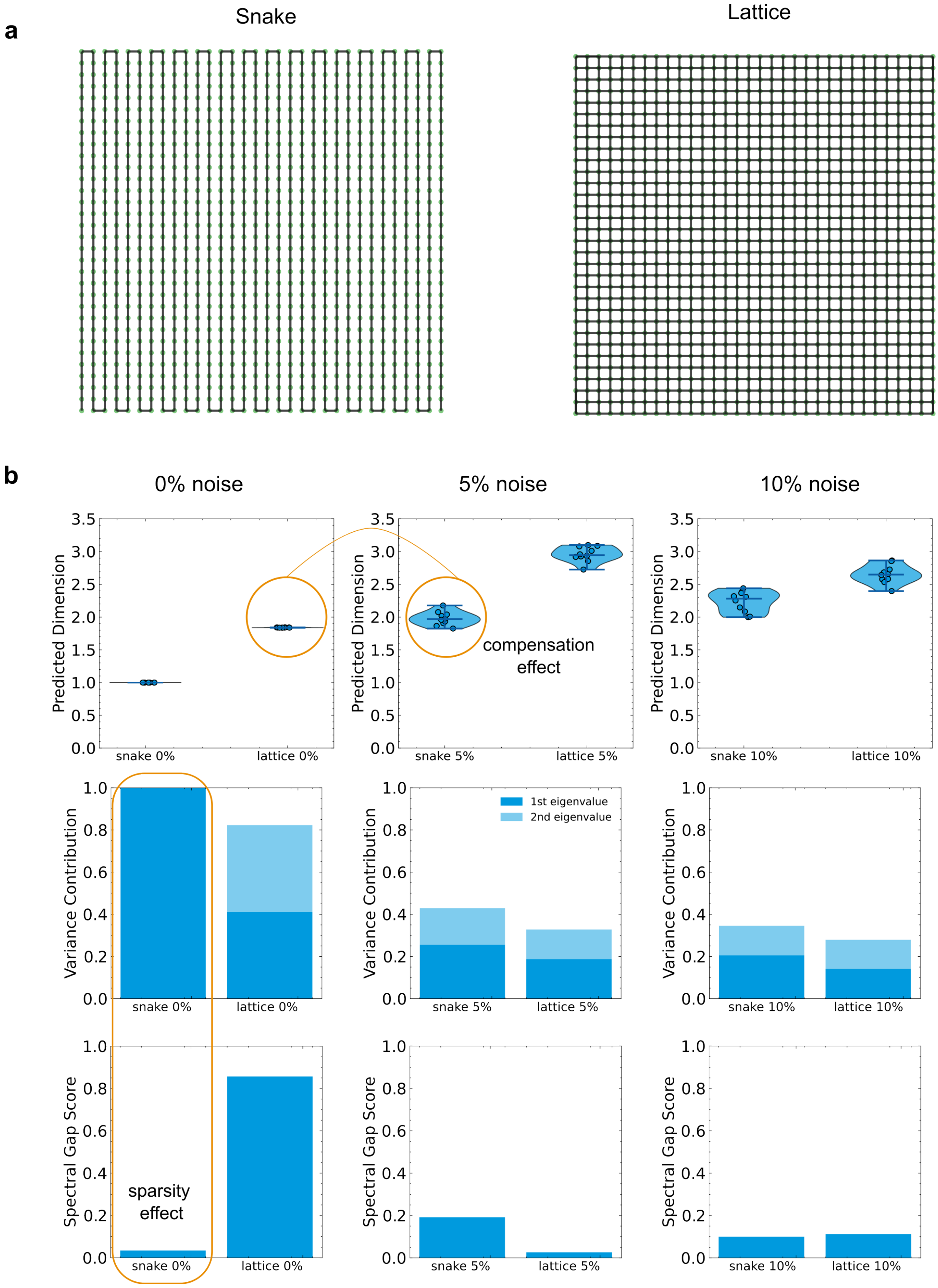

Figure S9: Compensation and sparsity effects in a toy example comparing snake-like lattice and square lattice topologies. **a** Node and edge distribution in physical space for both lattice types. **b** Predicted dimension under increasing noise levels. Moderate noise can make the one-dimensional snake lattice appear more two-dimensional. However, the spectral gap score remains low, indicating that the second and third eigenvalue are comparable, which is a consequence of the underlying one-dimensional ”chain” structure.

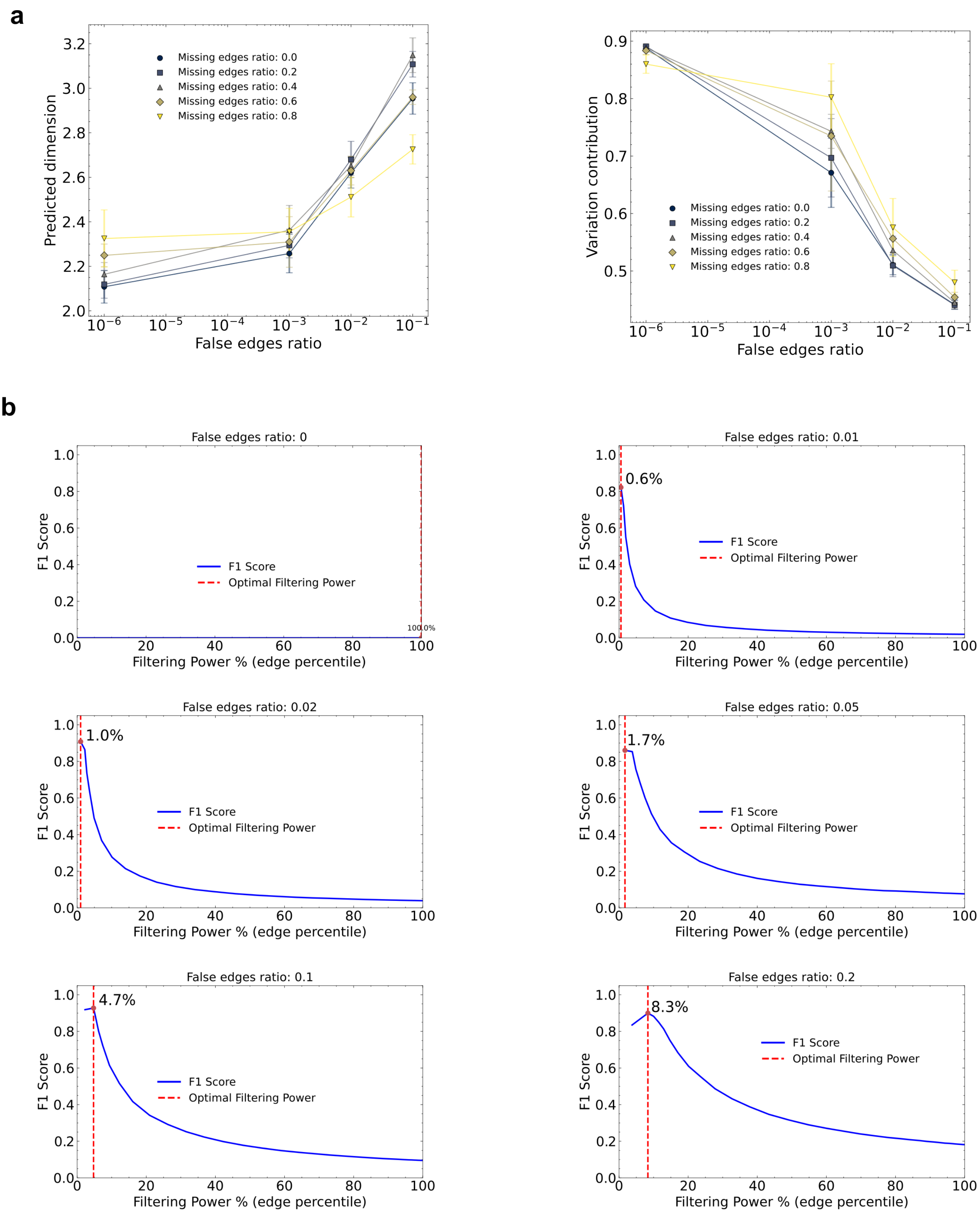

Figure S10: Supplement to the main figure panel Fig.3f. **a** Network dimension and Gram matrix variance contribution for graphs under different conditions of missing edges and false edges ratios. The experiment was repeated n=20 times for each data point to obtain a 95% confidence interval. **b** Quantitative evaluation of the optimal filtering power, according to the F1 score.

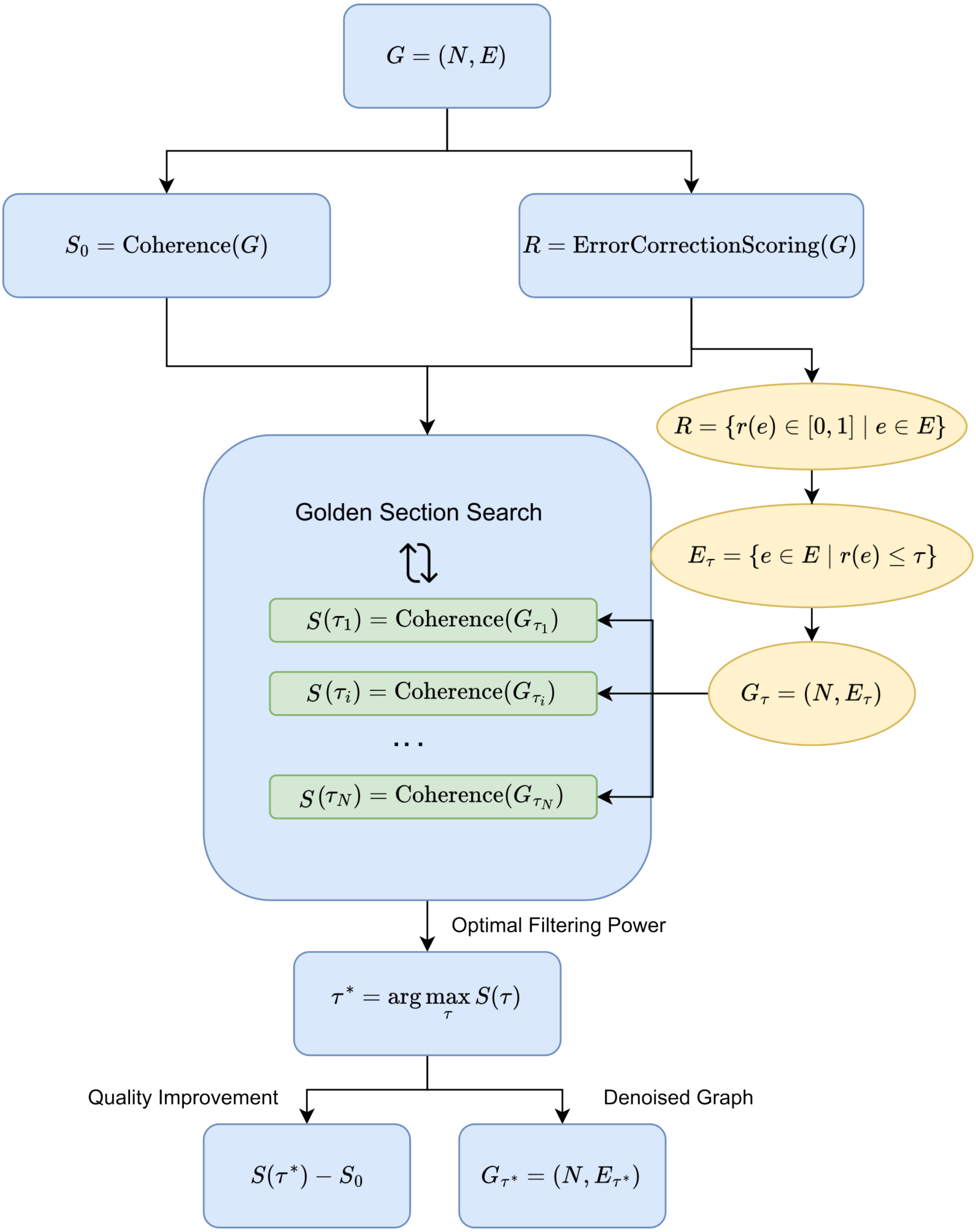

Figure S11: Filtering power optimization algorithm as a synergy between error correction strategies and spatial coherence metrics. Given a proximity graph $G = (N, E)$, a spatial coherence metric is computed S_0. Next, edge confidence scores are then assigned following an error correction strategy, producing a ranked list of edges R. A golden section search is used to identify the optimal filtering threshold τ^* that maximizes the spatial coherence of the filtered graph G_τ. The final output is the denoised graph G_{τ^*}, along with the improvement in spatial coherence $S(\tau^*) - S_0$.

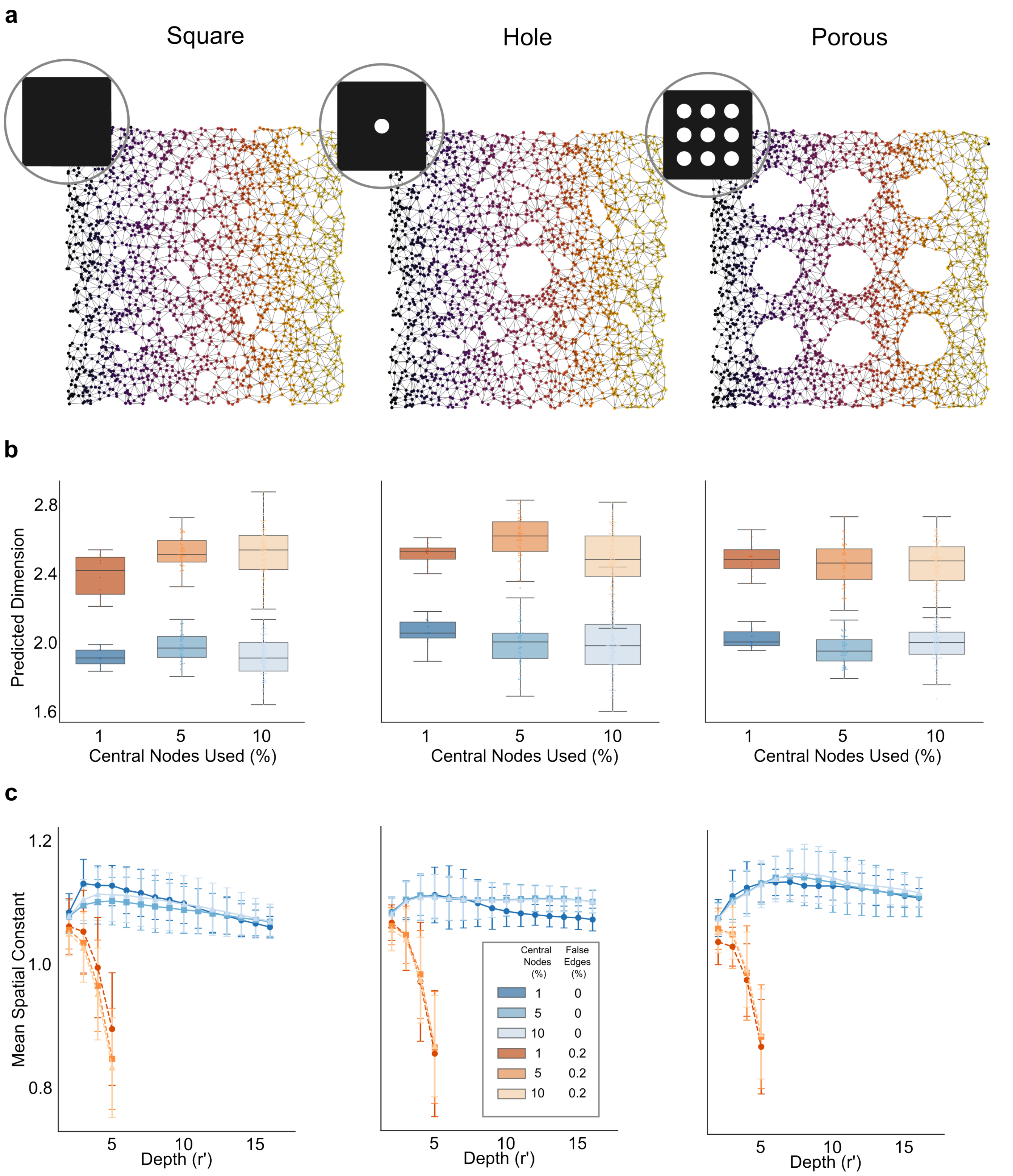

Figure S12: Effect of the number of central nodes on predicted dimension and spatial constant on usual and porous geometries. **a** Node and edge layouts in physical space for three geometries with varying porosity levels. **b** Predicted dimension for each geometry using different central node ratios (1%, 5%, and 10%) under two conditions: no noise and 0.2% noise. **c** Mean spatial constant under the same conditions and node ratios.
