# Supplementary material for: Spatial coherence in DNA barcode networks
Source: Patterns (N Y). 2025 Dec 1;6(12):101428. doi: 10.1016/j.patter.2025.101428 (PMC12745985; doi:10.1016/j.patter.2025.101428)
Supplement: Document S2. Article plus supplemental information [file mmc2.pdf]

Patterns

Article

Spatial coherence in DNA barcode networks

Highlights

- Improves understanding of what makes spatial networks reconstructable
- Develops quality control for sequencing-based microscopy with no extra experiments
- Detects incoherence from network data alone with three quantitative geometry measures
- Guides error-correction algorithms to maximize signal and minimize noise

Authors

David Fernandez Bonet, Johanna I. Blumenthal, Shuai Lang, Simon K. Dahlberg, Ian T. Hoffecker

Correspondence

ian.hoffecker@scilifelab.se

In brief

This study introduces spatial coherence, a way to check whether DNA barcode networks follow conventional geometry rules. Three geometry tests detect network quality, help with filtering corrections, and improve sequencing-based microscopy reconstructions in both simulations and published datasets.

Bonet et al., 2025, Patterns *6*, 101428
December 12, 2025 © 2025 The Author(s). Published by Elsevier Inc.
https://doi.org/10.1016/j.patter.2025.101428

CellPress

Patterns

CellPress
OPEN ACCESS

Article

Spatial coherence in DNA barcode networks

David Fernandez Bonet,[1] Johanna I. Blumenthal,[1] Shuai Lang,[1] Simon K. Dahlberg,[1] and Ian T. Hoffecker[1,2,*]
[1]Science for Life Laboratory, Department of Gene Technology, KTH Royal Institute of Technology, Tomtebodavägen 23a, 171 65 Solna, Sweden
[2]Lead contact
*Correspondence: ian.hoffecker@scilifelab.se
https://doi.org/10.1016/j.patter.2025.101428

THE BIGGER PICTURE Modern biology depends on simultaneously understanding both what kind of molecules are in a tissue and where they are. For example, knowing where molecules are in relation to each other can be key when trying to tell a healthy sample from a pathological sample. Sequencing-based microscopy provides an unconventional way to see this: instead of using light, it uses DNA. The approach comes with advantages such as scalability, reduced instrumentation, and easier 3D imaging. To achieve this, each molecule carries a short DNA barcode that interacts with nearby barcodes, forming a network. Reading and decoding this network can reconstruct the original layout of a tissue, providing information both on where molecules are and what kind of molecules they are.

But there can be small errors in the form of long interactions (instead of short ones) or small mismatches in the DNA barcodes. These errors are difficult to spot and can distort the resulting image. Therefore, a key effort in the field consists of building effective quality controls and detecting spurious interactions. Existing quality checks rely on comparing previously obtained images, but those are often unavailable or costly to obtain. The present work navigates around this by providing a direct quality control that does not require extra data. Over time, spatial coherence measures could become a shared benchmark for quality control and error correction used in different technologies, improving the accuracy of protein and transcript maps and having an impact on our understanding of both basic biology and disease diagnostics.

SUMMARY

DNA barcode networks are the basis of sequencing-based microscopy, an emerging family of chemical imaging methods aiming to reconstruct spatial information, without optics, using sequencing technology. These methods capture microscopic spatial information by forming networks composed of many local chemical interactions, each marked by a unique, DNA-based barcode. However, the fundamental laws governing such networks are not yet understood, and spatial barcode networks are influenced by structural distortions such as false or shortcut edges. Current methods lack ground-truth-free tools to validate spatial quality, and we address this with a framework for topology-based quality control. We define a fundamental feature of spatial networks, spatial coherence, which quantifies geometric self-consistency in a network. By formalizing this relationship into quantitative metrics adapted from classical geometric rules, we could quantify spatial distortions by using only network data and show how these can be used as an optimization criterion to iteratively improve spatial reconstruction.

INTRODUCTION

Advancements in imaging technologies enabling high-plex spatial molecular mapping have revolutionized our approach to studying complex systems such as molecular physiology, development, or pathology.[1,2] While optical imaging methods provide high-resolution images of multiple targets, single-cell methods[3,4] offer extensive gene data without spatial context. Spatial omics techniques[5–13] seek to balance this trade-off with high multiplicity coupled with spatial resolution. This approach depends on relating position to sequence information with spatial reference maps that are obtained via, e.g., microscopy-based *in situ* sequencing or deterministic assignment.

Sequencing-based microscopy,[14–23] in contrast, seeks to spatially resolve many molecular targets but without reliance on a spatial reference map. To do this, these methods use DNA barcode networks to capture spatial relationships between molecules, enabling recovery of molecular positions and image formation by sequencing and reconstructing the recorded spatial network. While individual techniques differ in terminology

This is an open access article under the CC BY license (http://creativecommons.org/licenses/by/4.0/).

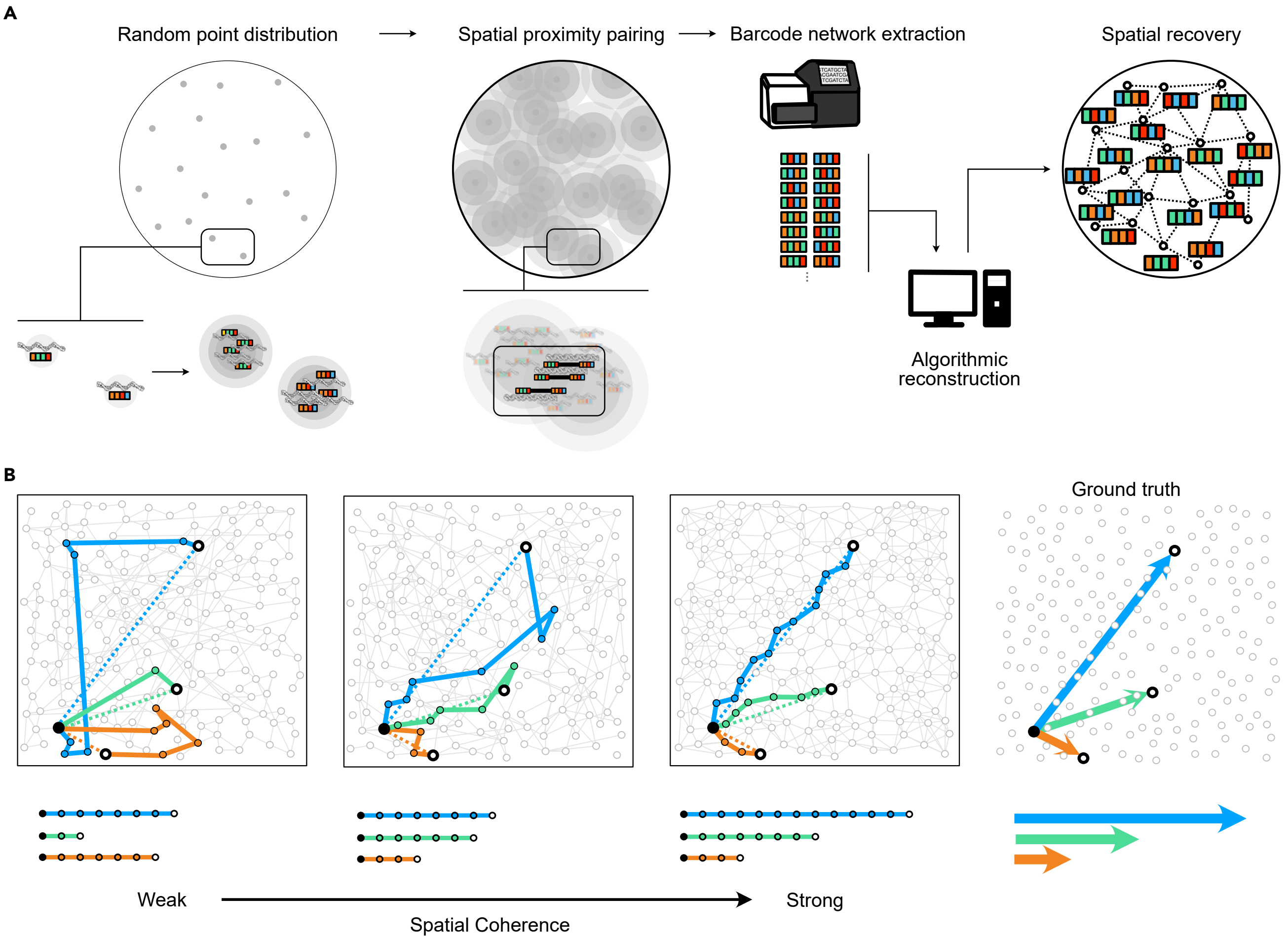

Figure 1. Spatial networks in sequencing-based microscopy

(A) Sequencing-based microscopy pipeline schematic. DNA strands are distributed across space, each carrying a random DNA barcode. Seeded strands are clonally amplified, forming polonies, each representing a region of space. Polonies link covalently, forming a proximity record. Strands are sequenced, converted to an edge list, and reconstructed into a spatial network whose structure preserves proximity relationships.

(B) A progression of networks of increasing spatial coherence, showing the Euclidean distance ground truth (far right) and shortest path network hop distances for each topology. Networks with low spatial coherence have the shortest path distances that correlate poorly with the ground-truth Euclidean distances, vs. a strong correlation for spatially coherent networks.

(e.g., "DNA microscopy," "molecular pixelation," etc.), they share a unifying structure: a spatial network formed by DNA barcodes or unique molecular identifiers (UMIs) whose connections reflect molecular proximity. We use the term DNA barcode network as a general descriptor of this class of spatially informative graphs. This "inside-out" approach comes with potential advantages, including molecular target multiplexing, scalability, reduced instrumentation, and isotropic imaging in 3D or optically opaque samples.

Sequencing-based microscopy methods (Figure 1A) localize molecules of interest, such as messenger RNA or proteins in biological samples, and tag them with unique DNA identifiers (barcodes). These barcoded sequences are amplified, forming local patches of space represented by a unique barcode sequence (polonies). Spatially adjacent polonies interact to form a covalent linkage and, at sufficiently high densities, form a proximity graph[24] (hereafter called a spatial network). While the precise molecular mechanism of barcode localization varies across techniques, e.g., via hybridization, reverse transcription, antibody conjugates, etc., the resulting structure is consistently a graph of molecular proximity encoded in DNA sequence form. This information is then recovered through sequencing and computational reconstruction of relative molecular positions. Sequencing-based microscopy has progressed from *in silico* models[14,18,20,21,25] to experimental realizations in 2D and 3D.[17,22,26]

However, assessing the quality of reconstructed images without prior knowledge of spatial positions remains a challenge. Current approaches to image validation in sequencing-based microscopy use comparison to alternatively sourced ground-truth or sample information, such as known biological patterns of co-localization,[22] or images from alternative microscopy modalities.[17,26] This practice forgoes the opportunity to access topological information that is common to all spatially constrained networks. Such networks exhibit what we term spatial coherence (Figure 1B), or the tendency for network distances to behave consistently according to the geodesics of a manifold. When the

CellPress
OPEN ACCESS

manifold is flat, this reduces to a correlation with Euclidean distances, but more generally, it reflects geometric self-consistency. As shown with the Swiss-roll benchmark (Figure 4), edges that do not follow the manifold's geodesics (even if short) can in fact violate this self-consistency and reduce coherence.

This perspective of spatial coherence connects network geometry and real-world geometry and provides a new way to quantify data reliability and improve reconstruction accuracy. We show that spatial coherence is a measurable feature of well-behaved spatial networks and, conversely, that low spatial coherence can indicate noise or distortions, independently of the subject being imaged. This implies a promising opportunity to at least partially validate reconstructed images in sequencing-based microscopy without prior sample knowledge. The spatial coherence framework refers to the suite of metrics we propose—network dimension, spatial constant, and spectral scores—to assess structural consistency of spatial graphs independently of reconstruction. All the methods presented in this study are implemented in a user-friendly package available publicly.

Topology-only indicators of network spatial coherence

Whereas certain real-world networks exhibit a small-world property in which the number of hops between two nodes is short compared to the network size,[27,28] spatial networks, in contrast, exhibit a "large-world" property where traversal between distant points in physical space takes correspondingly many network hops in network space.[29] This hop distance (known as the network or shortest path distance) and the Euclidean distance are correlated for networks formed according to consistently applied spatial constraints[18,25] (Figures 2A–2C). For example, in the European high-speed rail network, it takes more connections to reach Rome from Stockholm than from Copenhagen. However, to measure the consistency with which network distances obey such Euclidean-like constraints without comparison to ground-truth Euclidean data, we may instead use relative comparison between geometric quantities, which have counterparts in the context of spatial networks. We identified at least three such relationships: the spectral analysis of the Gram matrix, which contains information on the network's distances; the stability of average distances with respect to size (the spatial constant); and the intrinsic network dimension (methods: spectral analysis of the Gram matrix, network dimension, and spatial constant).

To quantify the intrinsic network dimension, we drew analogies to Euclidean scaling laws (Figures 2D and 2E). The relationship between the number of points within a $\mathcal{D}$-dimensional ball and its radius r follows a power law (Equation 1):

$$\frac{N}{C} = \alpha r^{\mathcal{D}}, \qquad \text{(Equation 1)}$$

where N is the number of points, C is the concentration or point density, and α is a geometric factor ($\alpha = \pi$ for a circle and $\alpha = \frac{4}{3}\pi$ for a sphere). We apply this to DNA barcode networks to infer the dimension of the network,[30] where N represents the number of moles, C represents the solution concentration, and the intrinsic network dimension $\mathcal{D}'$ is determined by Equation 2:

$$\frac{N}{\langle k \rangle} = \alpha r'^{\mathcal{D}'}. \qquad \text{(Equation 2)}$$

Here, we substitute the Euclidean radius r with the network distance r', which represents the depth of a breadth-first search (BFS) from a given origin node. N represents the nodes within the subgraph that fall within r' of this origin node, $\langle k \rangle$ denotes the average node degree, and α acts as a geometric factor. In a network where the topology is aligned with Euclidean geometry, the values for N and r' will obey a power law where the power $\mathcal{D}'$ corresponds to the original dimension of the physical space. Origin nodes from the BFS are selected according to their closeness centrality, with the empirically robust heuristic of using the top 1% nodes with a hard minimum of 10 and a soft maximum of 100 (Figure S12; methods: network dimension).

Another geometric scaling law relates to the size of the space and the average distance between its points.[31] This expected distance $\langle d \rangle$ in Euclidean space is linked to the space size and dimension $\mathcal{D}$ through a constant:

$$\beta = \langle d \rangle \left(\frac{C}{N}\right)^{\frac{1}{\mathcal{D}}}. \qquad \text{(Equation 3)}$$

Here, $\frac{C}{N}$ represents the size of the space, equivalent to volume in 3D or area in 2D. This dimensionless constant, β, remains consistent across scales in Euclidean space for a given dimensionality and boundary geometry (e.g., a square vs. a sphere). We extend this concept to network geometry with an analog dimensionless spatial constant $\mathcal{S}$:

$$\mathcal{S} = \langle d' \rangle \left(\frac{\langle k \rangle}{N}\right)^{\frac{1}{\mathcal{D}}}, \qquad \text{(Equation 4)}$$

where N is the number of nodes, $\langle d \rangle$ is replaced with the mean network distance $\langle d' \rangle$, and the point density (molar concentration) is substituted with the average node degree $\langle k \rangle$. Because this constant may be computed at different system sizes N (Equation 4), observing its stability across these scales is an indicator of the network obeying Euclidean geometry or being spatially coherent.

Finally, we explored a modified principal-component analysis (PCA) approach for spatial network data. We analyzed the proportion of Gram matrix eigenvalues to determine the contribution of variation along each expected spatial dimension, substituting Euclidean with network distances[32] (Figures 2F and 2G). In Euclidean space, the first $\mathcal{D}$ components account for all the variance, while the remaining dimensions do not contribute. Network distances whose first $\mathcal{D}$ Gram matrix eigenvalues represent a proportion of the eigenvalues close to 1 would thus represent high spatial coherence, i.e., consistency with Euclidean space constraints.

Additionally, we evaluated the empirical time complexity of calculating these three network properties simultaneously. The process scales sub-quadratically with the number of nodes (Figure S1), suggesting that the approach may be scalable across larger datasets.

RESULTS

Spatial incoherence coincides with distortion

We applied these metrics to assess spatial coherence in relevant variables affecting spatial networks and found that the measurements were consistent despite the architectural differences. In

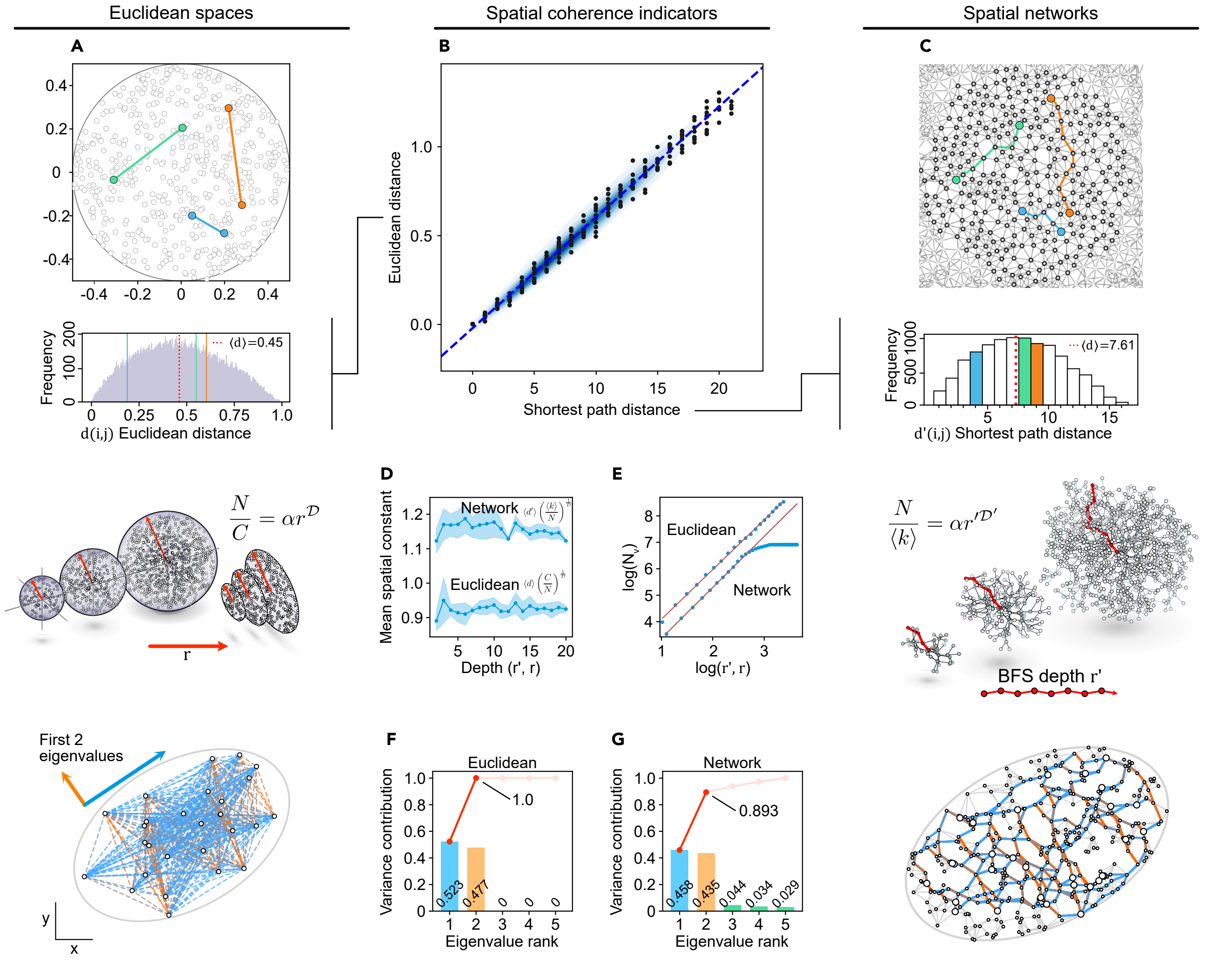

Figure 2. Geometric relationships for quantifying network spatial coherence

(A) Selected pairwise Euclidean distances between points on a disk and their frequency.

(B) Network shortest path distances and Euclidean distances are correlated in spatial networks.

(C) A spatial network is composed of edges forming paths between nodes, and the shortest paths are analogous to Euclidean pairwise distances, with a corresponding frequency distribution.

(D) Dimensionless spatial constant, relating pairwise Euclidean or shortest path distances to the volume of the space; it should remain constant with space size.

(E) A log-log plot of the scaling law relating Euclidean or breadth-first-search (BFS) radius to volume whose slope indicates intrinsic dimension of the space. BFS origin nodes are selected according to their closeness centrality, using nodes in the top 1% by closeness, with a hard minimum of 10 and a soft maximum of 100.

(F) The Gram matrix for a set of Euclidean points may be calculated from the pairwise distances of those points, the eigenvalues of which reveal the contributions to variation in the spread of points (left) from different dimensions (2 for spatially distributed points in 2D).

(G) The Gram matrix may also be computed from a set of shortest path distances, substituting for Euclidean distances in the case of a spatial network. The first eigenvalues, representing the number of dimensions of the space, should account for the variation in distances (right) in a spatially coherent network. Contributions from higher dimensions indicate variation that cannot be explained by spatial dimensions.

particular, we analyzed 2D, 3D, unipartite, bipartite, and diffusion networks; geographic networks (Figures S2 and S3); different network sizes (Figure S4); and shape and density variations (Figures S5 and S6). As expected for proximity-generated networks without structural noise, the spatial coherence metrics reported stable and consistent values.

In contrast, real data such as DNA barcode networks can contain false edges. These edges connect distant points in space and weaken the correlation between network distance and real-world distance. False edges may arise in sequencing-based microscopy via heterogeneous polony growth, PCR errors, or barcode collisions, resulting in indistinguishable identities. Such deviations reduce image reconstruction accuracy. An open challenge in sequencing-based microscopy is detecting and quantifying false edges without access to the ground truth. Therefore, we sought to demonstrate that the spatial coherence framework, which does not require a ground truth, can address this challenge.

To test how false edges affect spatial coherence, we progressively added them into an otherwise spatially coherent network

CellPress
OPEN ACCESS

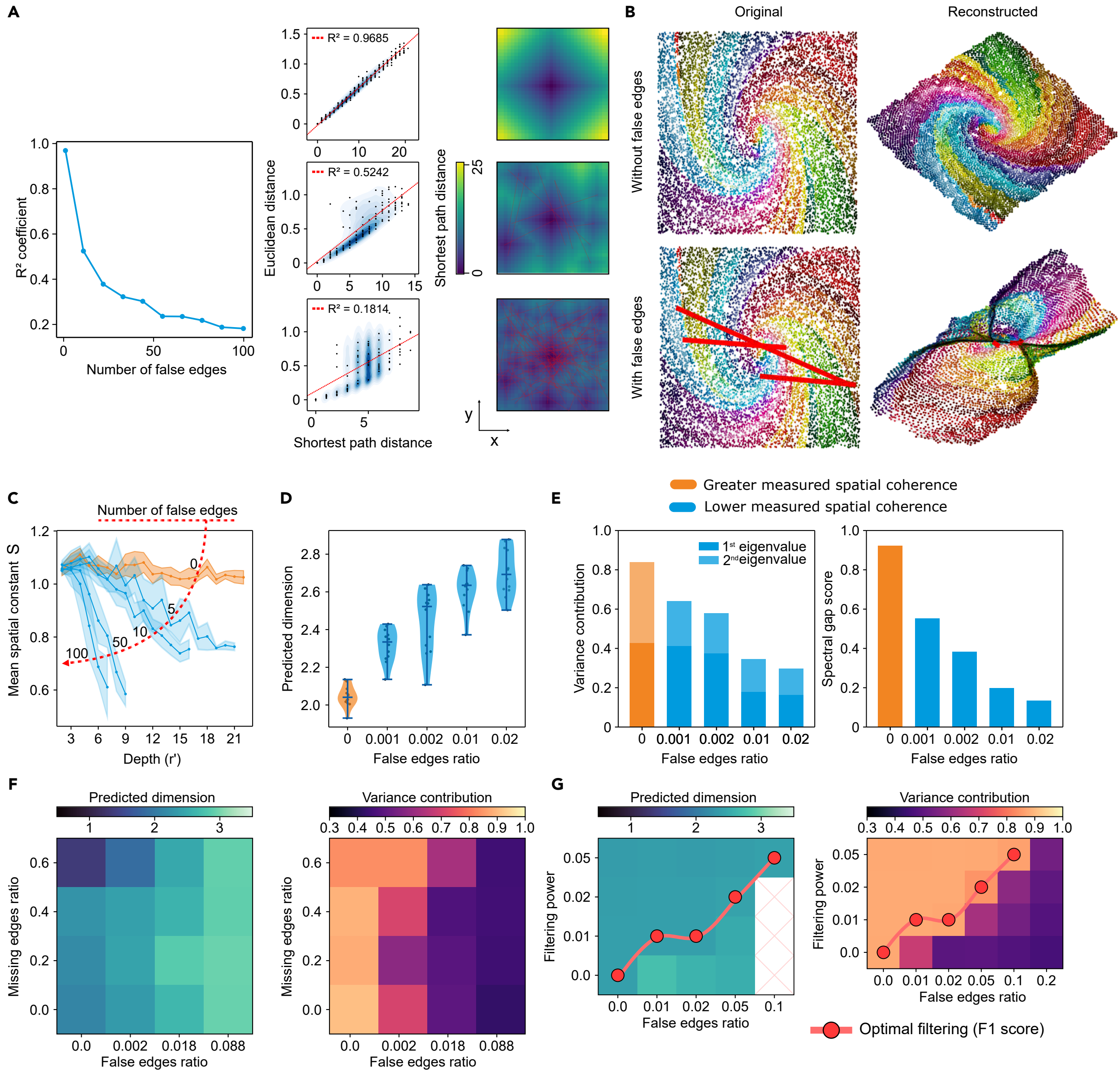

Figure 3. Impact of false edges in networks

(A) Coefficient of determination (R^2) showing the correlation between Euclidean and shortest path distance for an initially spatially coherent network across different levels of false edges. Three samples were selected to visualize such a correlation, along with the shortest path distance from the central node.

(B) Ground-truth original positions colored according to a spiral pattern and the reconstructed positions using network information only, followed by the same data with added false edges (highlighted in red) connecting distant regions and their corresponding reconstruction.

(C–E) Spatial coherence measures in response to progressively more false edges: (C) spatial constant values against BFS depth, (D) network dimension estimates, and (E) variance contribution of the first $\mathcal{D}$ eigenvalues and spectral gap scores.

(F) Effect of missing edges and error correction filtering on spatial coherence measures: dimension and Gram matrix variance contributions visualized across different false and missing edge frequencies.

(G) Dimension and Gram matrix variance contributions visualized for different false edge ratios and filtering powers, where the optimal filtering is highlighted for each case.

(Figure 3A). As false edges increased, the correlation between Euclidean and network distances decreased. This can be further visualized through the central node's network distances, which exhibit a radial progression in an optimal spatial network that is replaced by a random pattern as false edges are added. These artifacts influence the quality of reconstructed images (Figure 3B), for example, using the multidimensional scaling (MDS) algorithm (for results obtained with other algorithms, see

Figure S7). Here, false edges introduce conflicting distance constraints that require higher dimensions to resolve, analogous to folding a 2D sheet resulting in a 3D structure.

We next quantified these effects systematically with the spatial coherence framework (Figures 3C–3E). As false edges are progressively added to a well-behaved spatial network, the otherwise stable spatial constant profile drops as a function of depth, the network dimension (of a 2D physical space) increases from 2 to values near 3 (a similar behavior is observed for 1D rings[30]), and the variance and spectral gap scores decrease rapidly from the maximum score of 1. Furthermore, the length of the false edges also has an impact, with greater lengths leading to lower spatial coherence scores (Figure S8).

In contrast to false edges, missing edges could emerge from insufficient interactions or shallow sequencing, with sparseness reducing the spatial constraints necessary for accurate network reconstruction. Because experimental data could potentially have both false and missing edges, we investigated their combined impact on spatial coherence (Figure 3F). False edges generally increase the network's dimension and reduce the Gram matrix variance contribution, while missing edges lower the network dimension. In networks with 60% missing edges, the dimensionality approached 1. Notably, the combination of false and missing edges can show a compensatory effect: the right proportion can increase spatial coherence, with a dimension closer to two and higher variance contributions. However, this effect was only seen when the false edges ratio was low. While Figure S9 further expands on this in a case study with regular lattices, Figure S10A provides confidence intervals on these metrics by exhibiting several graphs under the same conditions and confirming that such intervals do not overlap for different noise levels.

We then applied a false edge correction algorithm to simulated data[33] (methods: error correction methods), measuring the spatial coherence at different filtering powers (Figure 3G). As the filtering power increased, more false edges were detected and eliminated but at the cost of also removing true edges. Indeed, the spatial coherence improved by showing a network dimension closer to two and a greater variance contribution for the principal Gram matrix eigenvalues. While a lower content of false edges required little filtering power to maintain coherence, higher ratios needed more stringent filtering. The optimal filtering power that maximizes the signal and minimizes noise can be understood with an F1 score and is shown in Figure S10B for every noise level. Given that the presence of false edges is generally unknown in experimental datasets, these results show that spatial coherence measurements allow us to optimize the filtering power to maximize false edge detection while minimizing the loss of true edges.

An important assumption in spatial coherence metrics is that physical space is globally Euclidean. But biological tissues often fold, branch, or mix 2D and 3D layers, potentially altering the geometric interpretation of network topology. To evaluate how such geometries affect the metrics, we use a Swiss-roll topology as a benchmark (Figure 4A). We compare three versions: a Swiss-roll with only proximity-based connections (SR), one with additional intermediate-length edges that connect nearby layers (ISR), and one with 10% random edges acting as noise (NSR).

Both the SR and ISR configurations represent a 2D manifold in 3D space. The spatial constants either increase or remain constant, and predicted dimension values are between 1.5 and 2 (Figure 4B). The variance contribution from the Gram matrix is high for SR (around 0.9), suggesting consistent geodesic structure. For ISR, the score drops (around 0.6), showing that short bridges connecting layers have a negative impact on the coherence. In contrast to SR and ISR, NSR shows a steep decline in the spatial constant, a predicted dimension close to 3 for a 2D manifold, and low variance contribution. These are indications that spatial coherence metrics are sensitive not just to the curvature of space but also to whether network distances align with meaningful geodesic structures.

At lower noise levels, however, the distinction becomes more subtle. Networks with lower noise containing random edges can still exhibit high variance contributions, comparable to ISR, making it harder to attribute structural variation to noise or true features. To understand this trade-off, we tested how much noise a Swiss roll can tolerate before it becomes clearly distinguishable from the ISR. As shown in Figure 4C, when the noise level is below 1% (false edge ratio < 0.01), the variance contribution remains relatively high. But after that threshold, the spatial coherence drops sharply, and noisy networks (NSR) become clearly separable from ISR. This suggests that for topologies with global curvature or a layered structure, intermediate variance contributions could potentially reflect biologically meaningful structures or low-level noises, depending on the context.

Spatial coherence is measurable in published datasets

We applied the spatial coherence framework to 2 published experimental datasets,[17,22] enabling us, according to the potential for reconstruction, to discriminate between individual graphs/specimens within the peripheral blood mononuclear cell (PBMC) dataset, as well as to identify a filtering threshold for the weighted network obtained by Weinstein et al. The human PBMC dataset contained hundreds of networks, and we performed spatial coherence measurements to benchmark it. We used the Gram matrix eigenvalues (Figure 5A) to select three representative networks that we reconstructed with the spatio-topological recovery by network discovery (STRND) algorithm.[25] Among these, PBMC 1 exhibited the best reconstruction, with short edges suggesting that the algorithm did not struggle to minimize edge lengths. PBMC 2 showed some longer edges, while PBMC 3 could not be reconstructed without several edge crossings. This quality gradient from PBMC1 to PBMC 3 could be attributed to the choice of reconstruction method, but the same gradient appeared independently with the spatial coherence measurements (Figures 5B–5D). This suggests that it is possible to predict if a network will reconstruct well even prior to reconstruction itself, which has utility when benchmarking large datasets. Matching reconstruction results, PBMC 1 displayed high spatial coherence in all measures. In contrast, PBMC 2 and PBMC 3 exhibited declining spatial constant profiles, variable network dimensions close to 3, and lower scores for the Gram matrix spectral analysis.

The second dataset consists of a weighted network that was filtered by different thresholds of interaction weight (Figure 5E). We hypothesized that greater weights would be related to more reliable interactions and corroborated that it indeed

CellPress
OPEN ACCESS

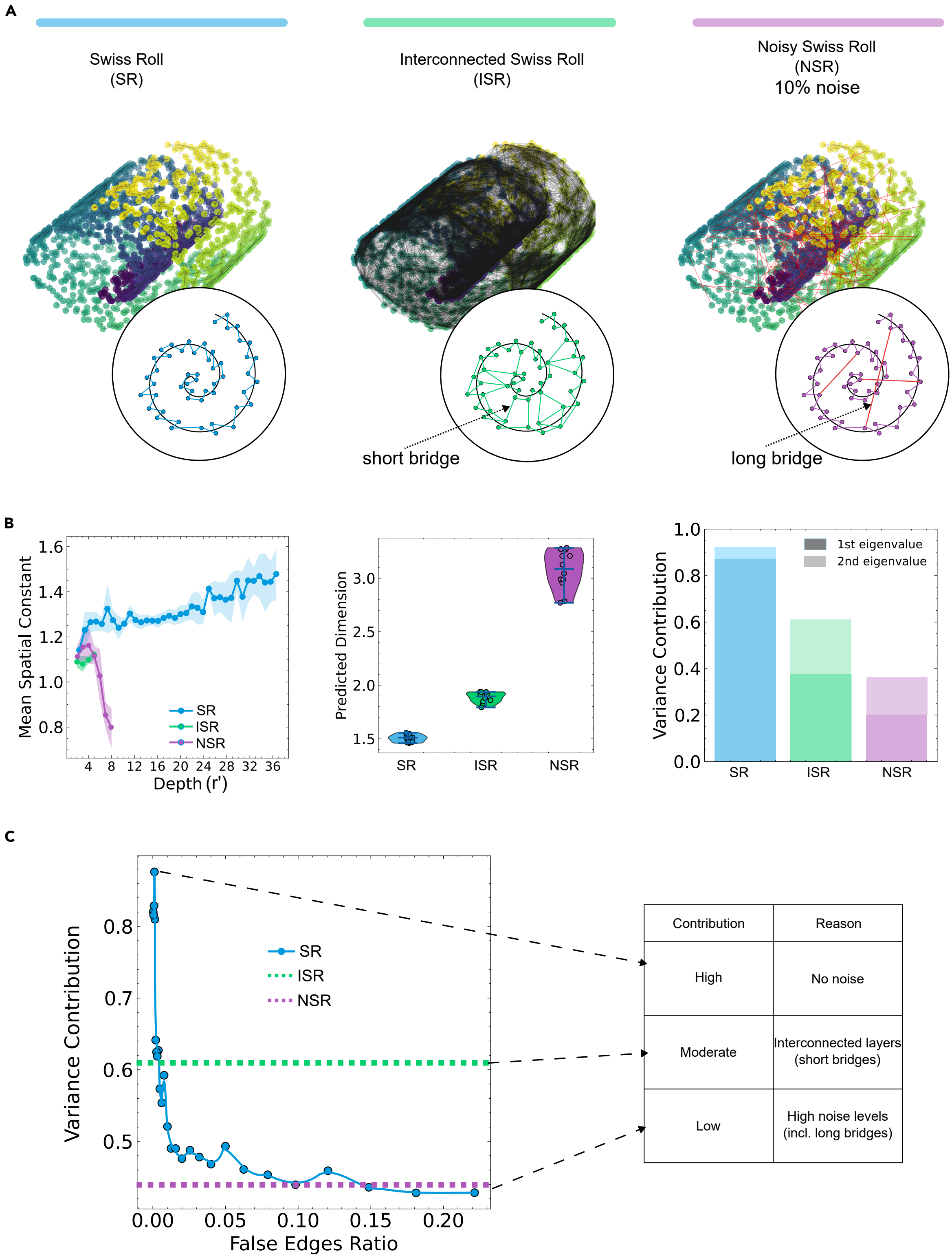

Figure 4. Spatial coherence on Swiss-roll topologies

(A) Node and edge distribution in physical space for a Swiss roll with proximity-based connections only (blue), proximity-plus-intermediate-length connections forming short inter-layer bridges (green), and a combination of proximity and noisy (10%) random connections (purple).

(B) Spatial constant, predicted dimension, and variance contribution from the Gram matrix for the three network types.

(C) Variance contribution across Swiss-roll networks with progressively increasing noise levels, with constant lines for the values of the interconnected Swiss roll and the Swiss roll with 10% noise.

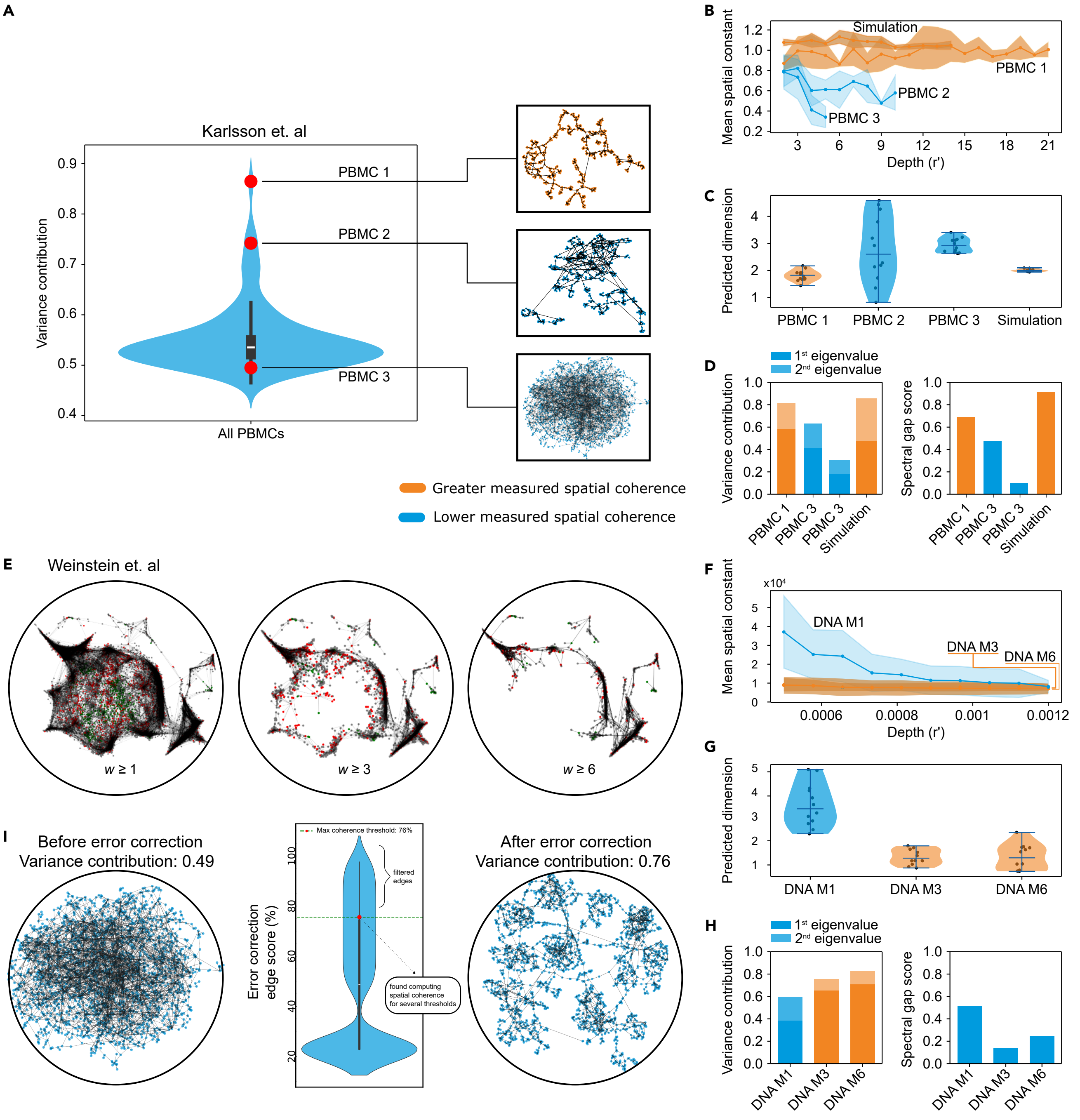

Figure 5. Spatial coherence measurement of published experimental data

(A) Variance contribution of the Gram matrix eigenvalues for the PBMC dataset from molecular pixelation, where 3 representative networks are selected and reconstructed.

(B–D) Spatial coherence measures for these representative networks and comparison to an ideal simulated profile: (B) spatial constant profiles for the representative networks, (C) predicted dimension measurements, and (D) spectral analysis, including variance contribution and spectral gap scores.

(E) Spatial coherence analysis of the DNA microscopy dataset: representative networks displayed using different weight filtering levels (with reconstructed locations taken from the spectral maximum-likelihood estimation result).

(F–H) Spatial coherence measures for different filtering levels of the DNA microscopy dataset: (F) spatial constant profiles, (G) network dimension, and (H) spectral analysis, including variance contribution and spectral gap scores.

(I) Error correction of an experimental network using spatial coherence metrics to inform the decision of threshold selection.

improves spatial coherence (Figures 5F–5H). With interactions that happened three or more times, the spatial constant stabilizes, and the network dimension decreases to values below 2. The variance contribution of the Gram matrix eigenvalues also gets closer to its maximum value of 1.

However, higher thresholds also result in sparser networks and lower-dimensional behavior and data loss. This is reflected in both the network dimension (values between 1 and 2) and the low contribution of the 2nd eigenvalue of the Gram matrix. The spectral gap score is also reduced, with the 3rd largest eigenvalue of comparable magnitude to the 2nd. Overall, similarly to simulated data, we see that harsher filters improve spatial coherence but also lead to data loss and sparser networks. Therefore, choosing an appropriate filter is important but difficult without prior knowledge. Here, we used simple count-based thresholds to isolate the effects of edge weight on coherence in an interpretable way. In this case, the spatial coherence measurements indicate that retaining interactions that occurred three or more times leads to networks that align better with Euclidean geometry. More sophisticated filters could offer performance benefits, and we note here that the spatial coherence framework is agnostic to filtering strategy and could serve as a feedback mechanism to guide and optimize such filters.

The lack of prior knowledge makes it difficult to select an appropriate filter that minimizes noise and maximizes signal. However, the spatial coherence metrics, which quantify noise, could be used as a parameter to systematically optimize the filter selection (Figure S11). We illustrate this in Figure 5I using a molecular pixelation network that initially exhibits low spatial coherence, with a variance contribution of 0.49. Edge confidence scores are assigned using an error correction method based on indirect path counts,[33] which favors edges between nodes with redundant connections. These scores are then used to guide filtering via the golden section search algorithm (methods: optimization of error correction filtering power), which efficiently identifies the threshold that maximizes the spatial coherence score. The violin plot (Figure 5I, middle) shows this threshold and the edge score distribution. After filtering, the updated network (Figure 5I, right) shows an improvement in spatial coherence, with the variance contribution increasing to 0.76, which can be seen visually as the corrected network displays mostly short, non-crossing edges. This is a limited example that demonstrates that spatial coherence metrics are not only useful for assessing network quality but can also be used as a natural tool for network denoising.

DISCUSSION

This study demonstrates that spatial coherence is a distinguishing feature of spatial networks, reflecting the correlation of real-world to network distances and thus the preservation of and possibility to detect and measure Euclidean-like geometric relationships in networks. This provides a way to evaluate quality in DNA barcode networks for sequencing-based microscopy, even without alternatively sourced ground-truth sample knowledge. Here, we note that while the interpretation of the predicted dimension metric assumes a weak model of spatial scaling (e.g., Euclidean-like distance growth), its measurement remains ground-truth free and is best interpreted as a relative measure of scaling coherence, particularly useful for detecting distortions in geodesic structure.

We show how spatial coherence can be measured across both simulated and published experimental networks and that it holds promise as a diagnostic tool for assessing data quality and detecting structural inconsistencies, such as false edges. To this end, we developed three simple measures to check if network distances obey Euclidean geometry: agreement between intrinsic network dimensions and physical dimensions, stability of the spatial constant, and spectral analysis of the network distance Gram matrix. These measures are general and may be broadly applied across present and coming technologies involving spatial networks. While the metrics presented here are empirically grounded and geometrically interpretable, we anticipate future work will formalize their theoretical basis, including the expected scaling behaviors under specific spatial generative models and connections to manifold learning theory.

An insight revealed by the Swiss-roll benchmark is the tension between Euclidean proximity and intrinsic geodesic structure. In the ISR variant, edges connect points that are nearby in the ambient 3D space but distant along the 2D manifold's surface. While such connections appear geometrically plausible in Euclidean terms, they violate the manifold's geodesic continuity, leading to reduced spatial coherence despite preserving short Euclidean distances. This reveals a fundamental paradox: proximity in the embedding space can conflict with topological fidelity. Spatial coherence metrics, particularly the spectral measures, are sensitive to this discrepancy, detecting distortion not from global curvature but from misaligned paths that shortcut the underlying geometry. This suggests a need to interpret coherence not as a proxy for strict Euclidean order but as a measure of geodesic consistency. Future extensions of this framework could benefit from explicitly incorporating manifold-aware geodesic models to better distinguish biological structures.

Beyond immediate applications in validating the quality of networks, spatial coherence could serve as an optimization criterion for error correction. As demonstrated in this study, correction algorithms can be improved by receiving feedback from spatial coherence measures, such as filtering power selection or potentially custom loss functions. This approach could reduce the reliance on extensive prior knowledge, providing a valuable but currently lacking quantifiable confidence measure for each individual network.

As spatial omics techniques evolve, the framework presented here could serve as a cost-effective method to evaluate DNA barcode networks, improve their quality, and provide validation metrics to the community.

METHODS

Spectral analysis of the Gram matrix

The rank of a Gram matrix derived from the pairwise distances of a set of points X is indicative of the dimension $\mathcal{D}$ of X. Therefore, the first $\mathcal{D}$ eigenvalues of the Gram matrix account for all the contributions, while the rest of the spectra have null values. In particular, a Gram matrix is defined by the dot product of its set of points X as $G = XX^T$. However, the point coordinates are not necessary to compute the Gram matrix, as it is also

related to the set's pairwise distances D (Equation 5), known as the Euclidean distance matrix (EDM)[34]:

$$D^2 = \mathrm{diag}\left(XX^T\right)\mathbf{1}^T - 2XX^T + \mathbf{1}\mathrm{diag}\left(XX^T\right)^T. \quad \text{(Equation 5)}$$

Here, 1 represents a column vector of all ones and diag$X$ denotes the column vector of the diagonal entries of the point coordinates matrix X.

This framework is generalized to networks, using the shortest path distance matrix (SPDM) D' as opposed to the EDM D. To obtain the network's Gram matrix $G = XX^T$, Equation 5 is simplified by using the geometric centering matrix J,

$$J = I_N - \frac{1}{N}\mathbf{1}_N\mathbf{1}_N^T, \quad \text{(Equation 6)}$$

where I_N is the $N \times N$ identity matrix and 1_N is an N-dimensional column vector of ones. Applying J results in positioning the centroid of X at the origin, effectively removing the terms diag$(XX^T)1^T$ and $\mathbf{1}\mathrm{diag}\left(XX^T\right)^T$ in Equation 5. This is known as double centering D'^2. Therefore, the Gram matrix can be obtained from D'^2 by applying Equation 7:

$$G = -\frac{1}{2}JD'^2J. \quad \text{(Equation 7)}$$

The eigenvalues of G coincide with the principal components obtained via PCA, up to a scaling factor.[32] Therefore, the magnitude of the eigenvalues is linked with the variance explained by each eigenvector. The normalized contribution of the first $\mathcal{D}$ eigenvalues $(\lambda_1, \ldots, \lambda_{\mathcal{D}})$ is expressed as

$$C_{\mathcal{D}} = \frac{\sum_{i=1}^{\mathcal{D}} \lambda_i}{\sum_{i=1} \lambda_j}. \quad \text{(Equation 8)}$$

This measure is bounded between 0 and 1 and reflects the extent to which the first $\mathcal{D}$ dimensions capture the variance of the data.

An additional measure is that of the spectral gap of the Gram matrix, defined as the normalized difference between the last $\mathcal{D}$ and the $\mathcal{D}+1$ eigenvalue:

$$\Delta\lambda = 1 - \frac{\lambda_{\mathcal{D}+1}}{\lambda_{\mathcal{D}}}, \quad \text{(Equation 9)}$$

where $\Delta\lambda$ denotes the spectral gap. A larger gap indicates a greater distinction between the relevant dimensions and the residual dimensions, with the largest gap occurring in a case where the residual dimensions take null values. This is in contrast to the variance contribution, which measures the total data variance captured by the first $\mathcal{D}$ eigenvalues without taking into account the rest of the eigenvalues. Figure S9 shows a case study where the variance contribution is high but the spectral gap is low.

Network dimension

The network's dimension is computed based on the Euclidean principle that the number of points (nodes) within a certain distance r increases according to a power law, where the exponent corresponds to the dimension of the space. To estimate the network's dimension, the shortest path distances d' act as proxies for Euclidean distances d. In a large spatially coherent network, the network dimension should agree with the Euclidean dimension of the space. However, boundary effects influence the measurement, making it dependent on the origin point and the depth of the scaling relationship interrogated at that point. If the measurement begins near a boundary, the perceived dimensionality will be lower compared to a central start.

Therefore, several central network nodes are identified in order to minimize finite-size effects. Centrality in our network analysis is defined by the closeness centrality measure, which identifies nodes that are in proximity to all other nodes in the network, thereby minimizing finite-size effects. Closeness centrality for a given node is calculated as the reciprocal of the sum of the shortest path distances from that node to all other nodes in the network. Mathematically, for a node i, the closeness centrality C_i is defined as

$$C_i = \frac{1}{\sum_{j=1}^{N} d'_{ij}}, \quad \text{(Equation 10)}$$

where $d'_i j$ is the shortest path distance between node i and node j and N is the total number of nodes in the network. Nodes with higher closeness centrality are used as starting points for a BFS of depth r', as they reduce boundary effects during the network traversal. The impact of the number of central nodes used is studied in Figure S12, where geometries with different porosity and noise levels are tested. Despite some fluctuations, the results across different proportions of central nodes are comparable even under different geometries and noise levels. Taking computational complexity into consideration, it is suggested to, as a rule of thumb, choose a default number of central nodes with closeness centrality in the top 1%, with a hard minimum of 10 nodes and a soft maximum of 100 nodes (whichever is smaller depending on graph size).

After the central nodes are identified, the number of nodes N within a depth r' is computed for all possible values of r', starting at $r' = 1$, where N represents the number of immediate neighbors one hop away from the origin. The gathered data are used to compute the fit $log(N)$ against $log(r')$ that enables finding the power defining the power-law behavior or the network's dimension. To ensure linearity, a sliding window of points is iteratively selected, and the segment providing the highest coefficient of determination is chosen. The slope of this linear fit is used to estimate the network's dimension, which, in the case of a spatial network, closely aligns with the dimensionality of the underlying Euclidean space.

Spatial constant

Analogous to the mean line segment in Euclidean space, the spatial constant $\mathcal{S}$ measurement is concerned with the mean distance between all pairs of nodes.

In Euclidean space, the mean line segment of a circle is[35]

$$\langle d \rangle \approx 0.9r, \quad \text{(Equation 11)}$$

where r is the radius of the circle. But $N = \rho V = \rho\pi r^2$, and Equation 11 becomes

$$\langle d \rangle = 0.9\left(\frac{N}{\pi\rho}\right)^{\frac{1}{2}}. \quad \text{(Equation 12)}$$

The spatial constant is chosen by definition as the dimensionless parameter $\mathcal{S} = \frac{0.9}{\sqrt{\pi}}$ that does not depend on density nor the

number of points. By rearranging the terms, it can be generalized to the network's case:

$$\mathcal{S} = \langle d' \rangle \left(\frac{\langle k \rangle}{N} \right)^{\frac{1}{\mathcal{D}}}, \quad \text{(Equation 13)}$$

where $\langle d' \rangle$ is the mean shortest path distance between nodes, N is the number of nodes, $\mathcal{D}$ is the network's original dimension, and $\langle k \rangle$ is the average degree of the network. The reason for using $\langle k \rangle$ as an analog of the Euclidean density ρ is that the average degree accounts for how many nodes there are on average at one space unit.

The assessment of the spatial constant's behavior is done by sampling the network at different size scales. First, origin nodes are selected at random, and a BFS traversal of fixed depth is conducted. This depth is increased iteratively, allowing for the discovery of more nodes and effectively increasing the size N. Different BFS depths represent different network sizes, reaching a maximum of half the diameter of the network on all instances. Therefore, this approach measures how the spatial constant changes with network growth. A consistent spatial constant that mirrors the behavior in Euclidean spaces would suggest the absence of false edges in the network. Conversely, a varying spatial constant indicates that the network's average shortest path length varies across different scales, pointing out that the behavior is not consistent and could potentially have false edges.

Algorithm implementation

All algorithms are implemented in Python 3.10, and the code is publicly available both as a GitHub repository and as a Python package. The implementation relies, at its core, on efficient network representations, shortest path distance computations, and BFS traversals.

This study takes advantage of sparse matrix representations for network data, specifically using the compressed sparse row (CSR) format. The choice is motivated by the efficiency in storing and manipulating sparse graphs, which have only a fraction of the potential $N(N-1)/2$ edges. Spatial networks are sparse in that sense because the number of edges is usually bounded by spatial constraints and scales proportionally to the number of nodes $N \propto E$. Using a CSR format can accelerate procedures to compute network properties, such as degree distribution and shortest path distance matrices. However, for the latter, they are memory intensive to store, as the space requirements scale quadratically with the number of nodes.

The SPDM D' denotes the shortest path distance D'_{ij} between nodes i and j. To compute the SPDM from the CSR representation of a network, we employ Dijkstra's algorithm, optimized for sparse matrices. In particular, for this work, the SPDM of a network is used to obtain the eigenvalues of its Gram matrix and to compute the network's dimension.

BFS is implemented by selecting an origin node and using a queue to keep track of the next nodes to visit. The depth of the traversal can be chosen to study the network at different scales, where higher depths result in bigger scales. Exploring different scales is used for the spatial constant computation, where several origin nodes are selected and BFS traversals with varying depths are run from them. This results in multiple subgraphs representing different network regions, each with its corresponding spatial constant.

In scenarios where the network's scale makes computations such as the shortest path matrix or eigenvalue decompositions prohibitively expensive, especially in terms of space complexity, the network can be sampled. This is done by selecting a starting node and running a BFS traversal until a certain number of nodes is reached. This threshold is user customizable and can be adapted depending on hardware.

Synthetic dataset

Networks are simulated by generating uniformly distributed points within a confined region, by default, a circle in 2D and a sphere in 3D. The geometric shape of these distributions primarily affects the boundaries and has a minimal impact on the bulk properties of the network. After generating points, networks are created based on the criterion that spatially close points form edges. Specifically, Figure S2 includes unipartite, bipartite, 2D, and 3D networks to model various experimental setups. Unipartite networks can form edges with all pairs of nodes, while bipartite networks consist of two distinct node sets where connections only occur between nodes from different sets. While existing research in sequencing-based microscopy predominantly uses 2D bipartite schemes,[17,22] our dataset also explores 3D networks due to growing interest in this modality.[26] Unless otherwise stated, the K-nearest neighbor (KNN) proximity is used with $K = 6$ in 2D and $K = 15$ in 3D, based on the average degree in Voronoi tessellations. Bipartite networks establish connections using the KNNs within their allowed node sets, ensuring no two nodes within the same set share an edge.

Furthermore, we also model connectivity via diffusion with an exponential distance decay in the interaction probability:

$$p_{ij} = \exp\left(- \frac{d_{ij}^2}{L_{\text{diff}}^2} \right), \quad \text{(Equation 14)}$$

where p_{ij} is the probability of interaction, d_{ij} is the pairwise distance, and L_{diff} is the diffusion length or the characteristic length scale of the exponential decay. The larger the diffusion length, the more likely interactions are between far-apart molecules.

For the specific experimental setting of DNA microscopy on diffusing amplicons,[17] the simulated dataset is obtained by running the pipeline at https://doi.org/10.5281/zenodo.10256692[33] with a default amplitude and spread of 10. The filtering algorithm from the same pipeline is used to study the spatial coherence before and after filtering.

False and missing edges

Modifications in the original network's topology are introduced in the form of false edges and missing edges. False edges are created by selecting pairs of nodes that are not originally connected and linking them. This is done until the desired false edge number or ratio is satisfied. Conversely, missing edges are created by removing existing edges. If the deletion disconnects the network, another pair of nodes is chosen. As with false edges, the process is iterated until the desired sparsity ratio is satisfied.

Error correction methods

A simple error correction strategy when the adjacency graph $G = (N,E)$ is weighted is to infer edge confidence directly from edge

weights. Larger weights had more node interactions, and this is interpreted as stronger evidence of physical proximity or adjacency. This is manifested in higher read counts or a greater number of concatenated barcoding events between molecules. For each edge $e \in E$, the confidence score is defined as

$$\text{score}(e) = w(e),$$

where $w(e)$ denotes the observed weight of edge e. Edges are ranked in descending order of weight and passed to the thresholding stage. This method is computationally efficient but depends on the weight distribution being varied in order not to discard too many edges.

An alternative strategy is adopted from indirect path counting.[33] In this method, each edge is assigned a score based on the number of indirect paths of a fixed length that connect its endpoints. The underlying assumption is that true spatial neighbors are typically embedded in a dense, locally connected neighborhood and thus are supported by many indirect paths, whereas spurious shortcuts often lack such redundancy. Specifically, for a candidate edge $e = (u,v)$, the score is defined as

$$\text{score}(e) = \#\{\text{indirect paths of length } L \text{ between } u \text{ and } v\}, \quad L = 3 \text{ by default.}$$

Direct edges are excluded from this count. High scores indicate stronger local support and increase the likelihood that the edge reflects genuine proximity. Low scores, in contrast, suggest that the edge may be spurious.

Both scoring approaches produce a ranked list of edges $R = \{e_1, e_2, \ldots, e_{|E|}\}$, ordered from most to least confident, which are subsequently used in the filtering optimization procedure.

Optimization of error correction filtering power

A denoised graph is obtained by removing spurious edges while preserving those that denote physical proximity. As the appropriate filtering power is generally unknown *a priori* (the amount of spurious edges is not known), a data-driven approach is adopted in which edge removal is guided by our spatial coherence metric. This approach is shown in detail in Figure S11. In particular, spatial coherence is quantified using the variance contribution of the first eigenvalues of the Gram matrix $C_{\mathcal{D}}$.

The initial spatial coherence score, denoted S_0, is computed for the unfiltered graph. Then, the ranked edge list R is obtained using the error correction strategy of choice.

To identify the optimal filtering threshold, a golden section search is performed to maximize the spatial coherence score $S(\tau)$, where $\tau \in [0,1]$ defines the proportion of edges to retain. The golden section search is an iterative method that locates the extremum of a unimodal function with minimal evaluations. At each iteration, two internal points within the current interval $[a,b]$ are selected:

$$x_1 = b - \frac{b-a}{\phi} \quad \text{and} \quad x_2 = a + \frac{b-a}{\phi},$$

where $\phi = \frac{1+\sqrt{5}}{2}$ is the golden ratio. The function is evaluated at both x_1 and x_2, and the interval is updated by retaining the half that contains the higher score. This process continues until a convergence criterion is met: either the minimum interval width is set to 0.01 or the maximum number of iterations is set to 50.

For each threshold τ, a filtered graph G_τ is constructed by retaining the top $\tau \cdot |E|$ edges from the ranking R. The updated Gram matrix is computed from G_τ, and the spatial coherence score $S(\tau)$ is evaluated. The threshold that maximizes spatial coherence is selected as

$$\tau^* = \text{argmax}_\tau S(\tau).$$

The final output consists of the filtered graph G_{τ^*}, along with the improvement in spatial coherence:

$$\Delta S = S(\tau^*) - S_0.$$

Experimental data acquisition

We applied the spatial coherence framework to 2 published experimental datasets: molecular pixelation[22] and DNA microscopy.[17] Both datasets consist of 2D bipartite networks but differ in their methodologies and target molecules. The molecular pixelation dataset focuses on the cell surface proteome, using rolling circle amplification to form DNA pixels that capture protein proximity. Nodes are referred to as pixels, and edges represent unweighted proximity interactions between protein antibodies and pixel products. In contrast, the DNA microscopy approach targets the transcriptome with a diffusion-based method to generate DNA polonies (nodes), weighting edges by interaction frequency to form weighted networks. Despite differences in data weighting and biological targets, both are compatible with the spatial coherence framework. While shortest path distances are used for unweighted molecular pixelation, a weight-to-distance transformation (methods: weighted networks) is used for the weighted DNA microscopy network.

The molecular pixelation PBMC dataset was downloaded from https://software.pixelgen.com/datasets/1k-human-pbmcs-v1.0-immunology-I/ and was processed according to the default guidelines (https://software.pixelgen.com/mpx-analysis/python/tutorials/introduction/). In particular, network components (putative cells) with 2,000 or fewer edges were not accounted for, and the metric τ, which measures the skewness of antibody counts, was used to identify outliers.[36] The representative networks PBMC 1, PBMC 2, and PBMC 3 correspond, respectively, to the networks originally named RCVCMP0001392, RCVCMP0002024, and RCVCMP0000120.

The DNA microscopy dataset was retrieved from https://www.ncbi.nlm.nih.gov/sra via accession number PRJNA487001, processed according to code from https://github.com/jaweinst/dnamic to perform read count filtration of 2 and deduplication based on edit distance, and then reconstructed into an image to reproduce the result of Weinstein et al.

Weighted networks

To simulate a weighted experimental setup where weights correspond to polony interactions, we follow the model proposed in previous studies,[17,33] which is based on Fick's law of diffusion. The model relates the reaction rate L_{diff} and the distance between two polonies, i and j, each of different types. This relationship is expressed by the equation

$$\omega(i,j) \propto t^{-\mathcal{D}/2} \times \exp\left(-\frac{\|x_i - x_j\|^2}{L_{\text{diff}}^2}\right), \quad \text{(Equation 15)}$$

where t represents the time since the start of the information transference step, $\mathcal{D}$ is the space dimensionality, and x_i and x_j are the coordinates of polonies i and j, respectively. The characteristic diffusion length L_{diff} is defined as

$$L_{\text{diff}} = \sqrt{8\mathcal{D}t}, \quad \text{(Equation 16)}$$

with D being the diffusion constant.

The diffusion constant value strongly depends on both the medium where DNA is diffusing and the length of the DNA strands. The variation can be several orders of magnitude. However, considering the known results for agarose gels (electrophoresis) with a 2% agarose concentration and a DNA strand length of about 10^2, D is roughly of the order $10^{-12}\frac{m^2}{s}$. Therefore, if the DNA strands are diffusing for some minutes, the order of magnitude of the characteristic length L will be about 10 μm.

Based on the diffusion model, the number of reactions between each pair of nodes can be estimated to determine the edge weights in the network:

$$w_{ij} = W \times \exp\left(-\frac{d_{ij}^2}{L_{\text{diff}}^2}\right), \quad \text{(Equation 17)}$$

where w_{ij} denotes the edge weight between nodes i and j, d_{ij} represents its corresponding distance, W is the amplitude reflecting the inherent reactivity of the polonies and sequencing depth, and L_{diff} indicates the effective interaction range via diffusion. The amplitude affects the likelihood and intensity of reactions, thereby influencing the weight of the connections in the resulting network graph.

Analyzing the relationship between the interaction range L_{diff} and the inferred distances, we derive the following from Equation 17:

$$d_{ij} = \frac{\left(-\ln\frac{w_{ij}}{W}\right)^{1/2}}{L_{\text{diff}}}. \quad \text{(Equation 18)}$$

The equation indicates that changes in L_{diff} scale the computed distance d_{ij} without altering the relative distribution among different pair distances. Consequently, variations in L_{diff} act as scale modifiers and do not impact the normalized or relative spatial distribution of node distances. Therefore, the interaction range is not a critical parameter for inferring the topology of the network in this model. Its primary role is establishing the scale of measurement rather than influencing the actual spatial configuration. As such, the model primarily relies on the amplitude W, which determines the reaction rate and can be found experimentally from peak weight observations.

RESOURCE AVAILABILITY

Lead contact

Requests for further information and resources should be directed to and will be fulfilled by the lead contact, Ian T. Hoffecker (ian.hoffecker@scilifelab.se).

Materials availability

This study did not generate new materials.

Data and code availability

- The molecular pixelation dataset[22] can be accessed at https://software.pixelgen.com/datasets/1k-human-pbmcs-v1.0-immunology-I/. The DNA microscopy dataset[17] can be accessed via the Sequencing Read Archive (project no. PRJNA487001, sample 3) at https://www.ncbi.nlm.nih.gov/sra/SRX5012191[accn. All other data reported in this paper will be shared by the lead contact upon request.
- All code necessary to reproduce the results in this paper is available through GitHub (https://github.com/DavidFernandezBonet/Network_Spatial_Coherence) and Zenodo (https://zenodo.org/records/15387529),[37] which also includes raw and preprocessed datasets.
- Any additional information required to reanalyze the data reported in this paper is available from the lead contact upon request.

ACKNOWLEDGMENTS

We acknowledge support from the Swedish Research Council (no. 2020-05368 to I.T.H.) and the European Research Council (ERC; nos. 949624 and 101138356 to I.T.H.). The authors wish to thank Erik Benson (Karolinska Institutet), Antti Elonen and Pekka Orponen (Aalto University), and Ragnar Thobaben (KTH Royal Institute of Technology) for helpful discussions and insights.

AUTHOR CONTRIBUTIONS

D.F.B. implemented the algorithms, characterization, and computational exploration in the study. J.I.B. and S.L. retrieved and analyzed data from the literature and ran the algorithms. All authors contributed conceptual insights. D.F.B., I.T.H., and S.K.D. wrote the manuscript. D.F.B. and I.T.H. conceived the study.

DECLARATION OF INTERESTS

I.T.H. is a scientific advisor to and holds equity in a privately held startup that develops technologies related to sequencing-based inference.

SUPPLEMENTAL INFORMATION

Supplemental information can be found online at https://doi.org/10.1016/j.patter.2025.101428.

Received: May 6, 2025
Revised: August 7, 2025
Accepted: November 3, 2025
Published: December 1, 2025

REFERENCES

1. Lewis, S.M., Asselin-Labat, M.L., Nguyen, Q., Berthelet, J., Tan, X., Wimmer, V.C., Merino, D., Rogers, K.L., and Naik, S.H. (2021). Spatial omics and multiplexed imaging to explore cancer biology. Nat. Methods *18*, 997–1012.
2. Moses, L., and Pachter, L. (2022). Museum of spatial transcriptomics. Nat. Methods *19*, 534–546.
3. Shapiro, E., Biezuner, T., and Linnarsson, S. (2013). Single-cell sequencing-based technologies will revolutionize whole-organism science. Nat. Rev. Genet. *14*, 618–630.
4. Gawad, C., Koh, W., and Quake, S.R. (2016). Single-cell genome sequencing: current state of the science. Nat. Rev. Genet. *17*, 175–188.
5. Ke, R., Mignardi, M., Pacureanu, A., Svedlund, J., Botling, J., Wählby, C., and Nilsson, M. (2013). In situ sequencing for rna analysis in preserved tissue and cells. Nat. Methods *10*, 857–860.
6. Lee, J.H., Daugharthy, E.R., Scheiman, J., Kalhor, R., Ferrante, T.C., Terry, R., Turczyk, B.M., Yang, J.L., Lee, H.S., Aach, J., et al. (2015). Fluorescent in situ sequencing (fisseq) of rna for gene expression profiling in intact cells and tissues. Nat. Protoc. *10*, 442–458.
7. Ståhl, P.L., Salmén, F., Vickovic, S., Lundmark, A., Navarro, J.F., Magnusson, J., Giacomello, S., Asp, M., Westholm, J.O., Huss, M.,

et al. (2016). Visualization and analysis of gene expression in tissue sections by spatial transcriptomics. Science *353*, 78–82.

8. Wang, G., Moffitt, J.R., and Zhuang, X. (2018). Multiplexed imaging of high-density libraries of rnas with merfish and expansion microscopy. Sci. Rep. *8*, 4847.
9. Karaiskos, N., Wahle, P., Alles, J., Boltengagen, A., Ayoub, S., Kipar, C., Kocks, C., Rajewsky, N., and Zinzen, R.P. (2017). The drosophila embryo at single-cell transcriptome resolution. Science *358*, 194–199.
10. Satija, R., Farrell, J.A., Gennert, D., Schier, A.F., and Regev, A. (2015). Spatial reconstruction of single-cell gene expression data. Nat. Biotechnol. *33*, 495–502.
11. Achim, K., Pettit, J.B., Saraiva, L.R., Gavriouchkina, D., Larsson, T., Arendt, D., and Marioni, J.C. (2015). High-throughput spatial mapping of single-cell rna-seq data to tissue of origin. Nat. Biotechnol. *33*, 503–509.
12. Halpern, K.B., Shenhav, R., Matcovitch-Natan, O., Toth, B., Lemze, D., Golan, M., Massasa, E.E., Baydatch, S., Landen, S., Moor, A.E., et al. (2017). Single-cell spatial reconstruction reveals global division of labour in the mammalian liver. Nature *542*, 352–356.
13. Rodriques, S.G., Stickels, R.R., Goeva, A., Martin, C.A., Murray, E., Vanderburg, C.R., Welch, J., Chen, L.M., Chen, F., and Macosko, E.Z. (2019). Slide-seq: A scalable technology for measuring genome-wide expression at high spatial resolution. Science *363*, 1463–1467.
14. Glaser, J.I., Zamft, B.M., Church, G.M., and Kording, K.P. (2015). Puzzle imaging: Using large-scale dimensionality reduction algorithms for localization. PLoS One *10*, e0131593.
15. Schaus, T.E., Woo, S., Xuan, F., Chen, X., and Yin, P. (2017). A dna nanoscope via auto-cycling proximity recording. Nat. Commun. *8*, 1–9.
16. Boulgakov, A.A., Xiong, E., Bhadra, S., Ellington, A.D., and Marcotte, E.M. (2018). From space to sequence and back again: Iterative DNA proximity ligation and its applications to DNA-based imaging. Preprint at bioRxiv. https://doi.org/10.1101/470211.
17. Weinstein, J.A., Regev, A., and Zhang, F. (2019). Dna microscopy: optics-free spatio-genetic imaging by a stand-alone chemical reaction. Cell *178*, 229–241.e16.
18. Hoffecker, I.T., Yang, Y., Bernardinelli, G., Orponen, P., and Högberg, B. (2019). A computational framework for dna sequencing microscopy. Proc. Natl. Acad. Sci. USA *116*, 19282–19287.
19. Gopalkrishnan, N., Punthambaker, S., Schaus, T.E., Church, G.M., and Yin, P. (2020). A DNA Nanoscope that Identifies and Precisely Localizes over a Hundred Unique Molecular Features with Nanometer Accuracy. Preprint at bioRxiv. https://doi.org/10.1101/2020.08.27.271072.
20. Boulgakov, A.A., Ellington, A.D., and Marcotte, E.M. (2020). Bringing microscopy-by-sequencing into view. Trends Biotechnol. *38*, 154–162.
21. Greenstreet, L., Afanassiev, A., Kijima, Y., Heitz, M., Ishiguro, S., King, S., Yachie, N., and Schiebinger, G. (2022). The DNA-based global positioning system—a theoretical framework for large-scale spatial genomics. Preprint at bioRxiv. https://doi.org/10.1101/2022.03.22.485380.
22. Karlsson, F., Kallas, T., Thiagarajan, D., Karlsson, M., Schweitzer, M., Navarro, J.F., Leijonancker, L., Geny, S., Pettersson, E., Rhomberg-Kauert, J., et al. (2024). Molecular pixelation: spatial proteomics of single cells by sequencing. Nat. Methods *21*, 1044–1052.
23. Dahlberg, S.K., Bonet, D.F., Franzén, L., Ståhl, P.L., and Hoffecker, I.T. (2025). Hidden network preserved in slide-tags data allows reference-free spatial reconstruction. Nat. Commun. *16*, 9652.
24. Mathieson, L., and Moscato, P. (2019). An introduction to proximity graphs. In Business and Consumer Analytics: New Ideas, pp. 213–233.
25. Fernandez Bonet, D., and Hoffecker, I.T. (2023). Image recovery from unknown network mechanisms for dna sequencing-based microscopy. Nanoscale *15*, 8153–8157.
26. Qian, N., and Weinstein, J.A. (2023). Volumetric imaging of an intact organism by a distributed molecular network. Preprint at bioRxiv. https://doi.org/10.1101/2023.08.11.553025.
27. Watts, D.J., and Strogatz, S.H. (1998). Collective dynamics of ‘small-world’networks. Nature *393*, 440–442.
28. Barrat, A., and Weigt, M. (2000). On the properties of small-world network models. Eur. Phys. J. B *13*, 547–560.
29. Zemel, R., and Carreira-Perpiñán, M. (2004). Proximity graphs for clustering and manifold learning. Adv. Neural Inf. Process. Syst. *17*, 225–232.
30. Shanker, O. (2007). Defining dimension of a complex network. Mod. Phys. Lett. B *21*, 321–326.
31. Parker, P., and Cowan, R. (1976). Some properties of line segment processes. J. Appl. Probab. *13*, 96–107.
32. Hastie, T., Tibshirani, R., and Friedman, J.H. (2009). The Elements of Statistical Learning: Data Mining, Inference, and Prediction, *2* (Springer).
33. Kloosterman, A., Baars, I., and Högberg, B. (2024). An error correction strategy for image reconstruction by DNA sequencing microscopy. Nat. Computat. Sci. *4*, 1–9.
34. Dokmanic, I., Parhizkar, R., Ranieri, J., and Vetterli, M. (2015). Euclidean distance matrices: essential theory, algorithms, and applications. IEEE Signal Process. Mag. *32*, 12–30.
35. Solomon, H. (1978). Geometric Probability (SIAM).
36. Yanai, I., Benjamin, H., Shmoish, M., Chalifa-Caspi, V., Shklar, M., Ophir, R., Bar-Even, A., Horn-Saban, S., Safran, M., Domany, E., et al. (2005). Genome-wide midrange transcription profiles reveal expression level relationships in human tissue specification. Bioinformatics *21*, 650–659.
37. Fernández Bonet, D. (2025). Network spatial coherence. Zenodo. https://doi.org/10.5281/zenodo.15387529.

Patterns, Volume *6*

Supplemental information

Spatial coherence in DNA barcode networks

David Fernandez Bonet, Johanna I. Blumenthal, Shuai Lang, Simon K. Dahlberg, and Ian T. Hoffecker

Supplemental Figures

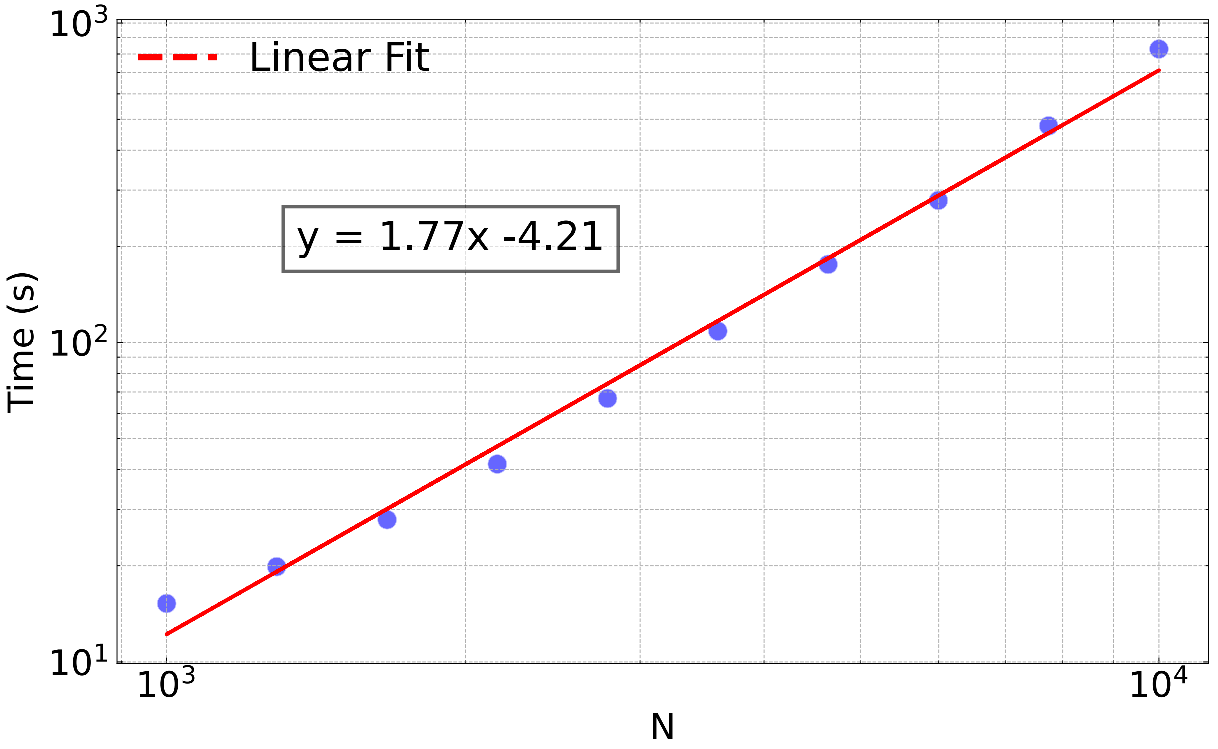

Figure S1: Empirical analysis of the time complexity associated with the spatial coherence pipeline. Computational time scales with varying input graph sizes, with a sub-quadratic scaling of 1.77.

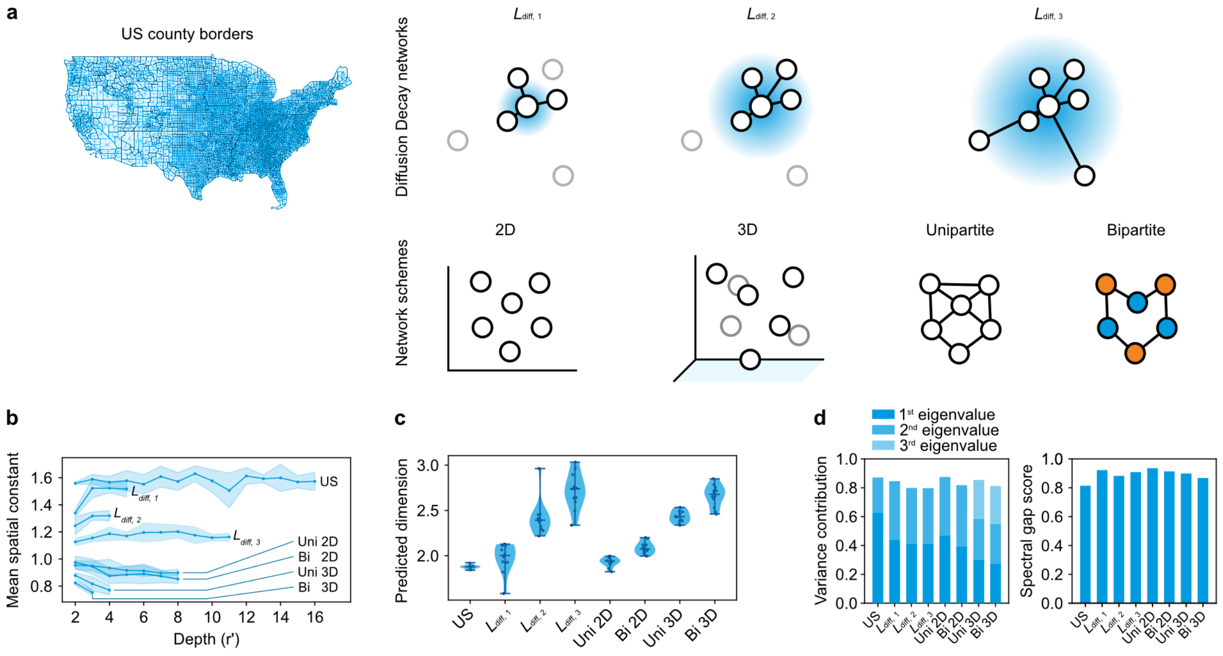

Figure S2: Spatial coherence is detectable in the topology of spatial networks (a) Different network modalities, including 2D/3D networks, unipartite/bipartite networks, and a distance decay based on diffusion to model interaction probabilities. Additionally, the US county borders network is included as a case study. (b) Spatial constant against depth revealing stable profiles for the spatial networks examined. (c) Network dimension values and (d) spectral analysis of the Gram matrix: variance contribution and spectral gap score showing values for spatial networks approaching 1.

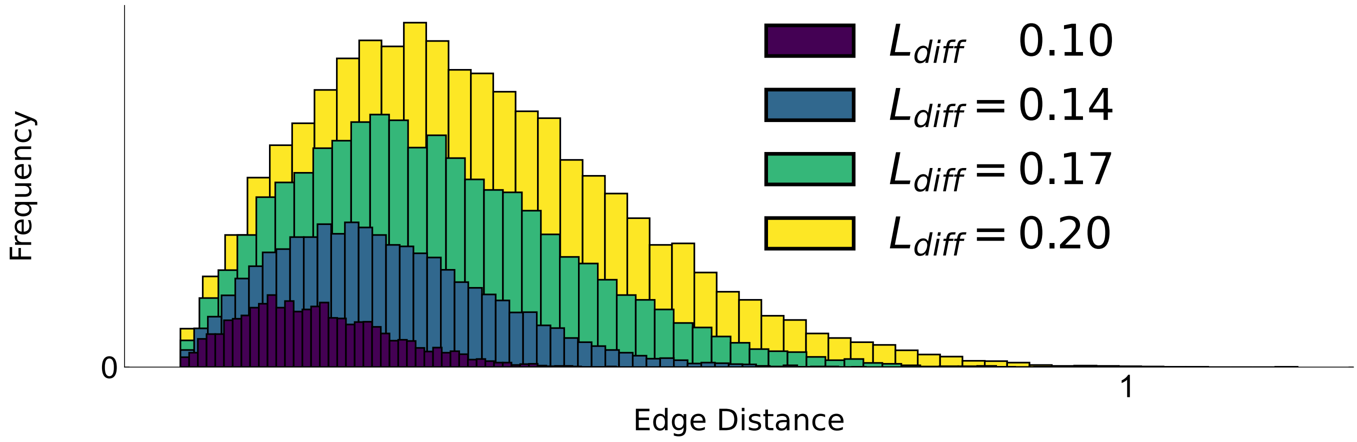

Figure S3: Edge length distribution for different characteristic diffusion lengths L_{diff}, used for network diffusion modeling.

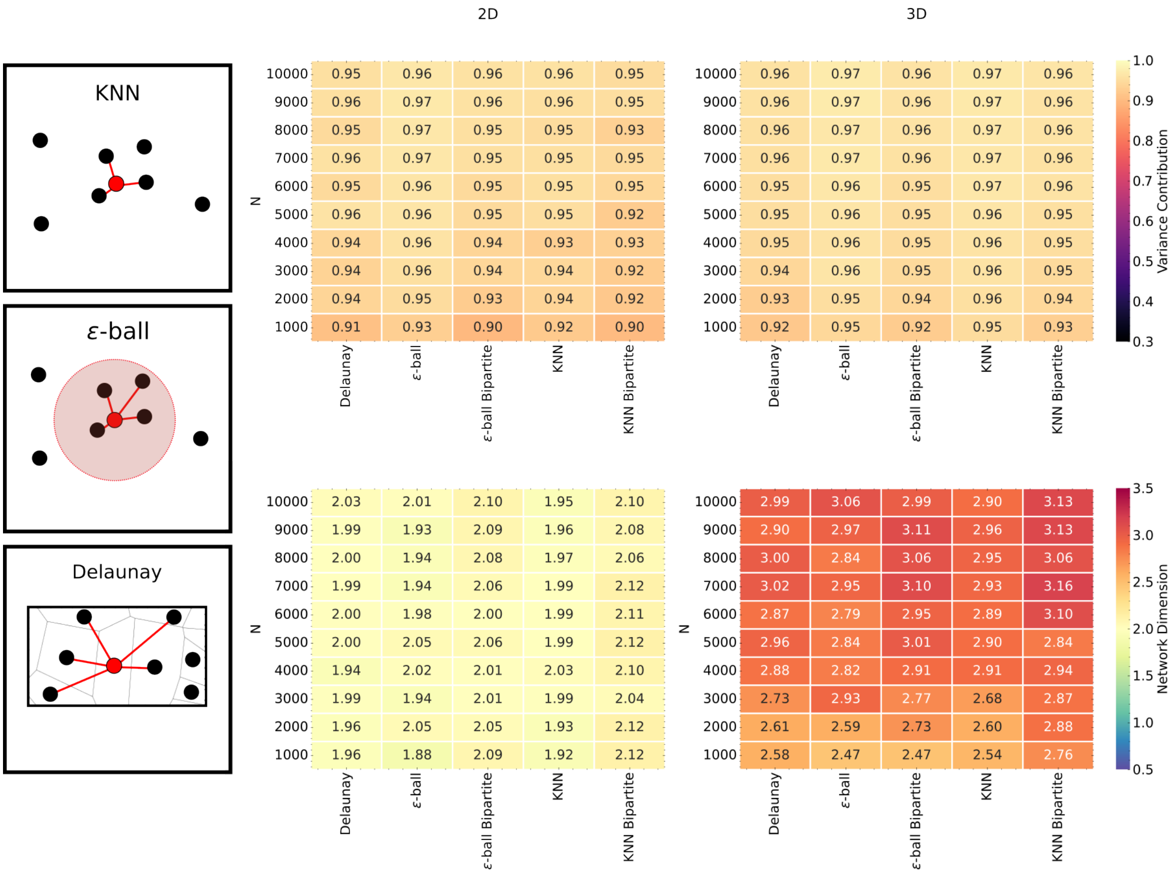

Figure S4: Spatial coherence measures analyzed across different numbers of nodes and proximity graph modalities in both 2D and 3D configurations, showing consistent results despite topological differences.

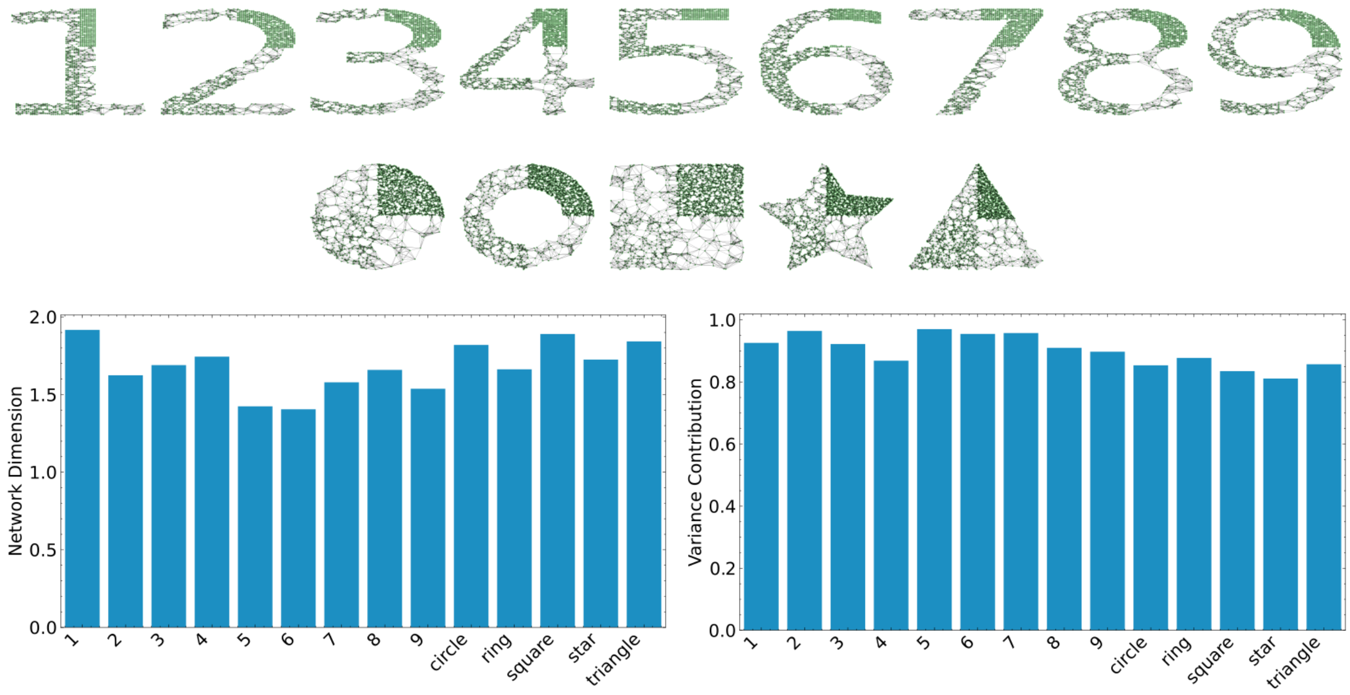

Figure S5: Spatial coherence measures on point clouds with different shapes, with variation regarding network dimension for shapes that are closer to a one-dimensional line and consistency for the variance contribution of the Gram matrix.

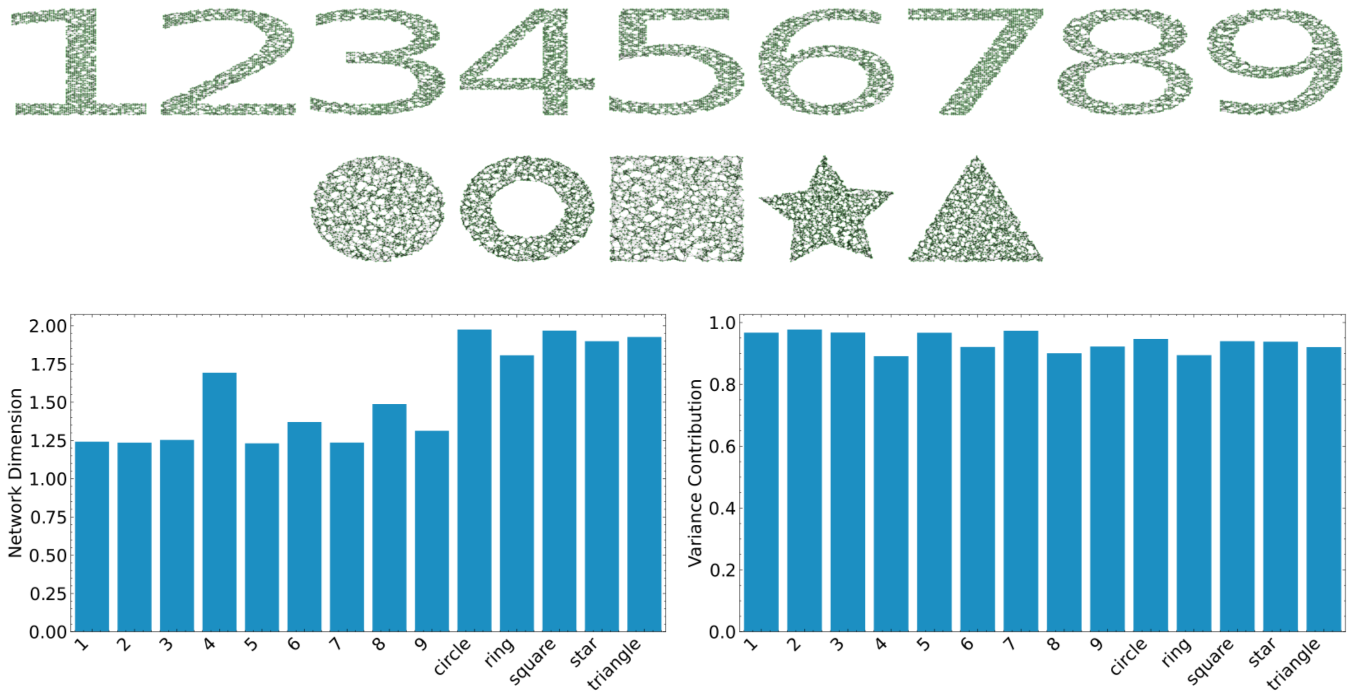

Figure S6: Spatial coherence measures on point clouds with different shapes and varying density regions, showing consistent values for spatial coherence measurements.

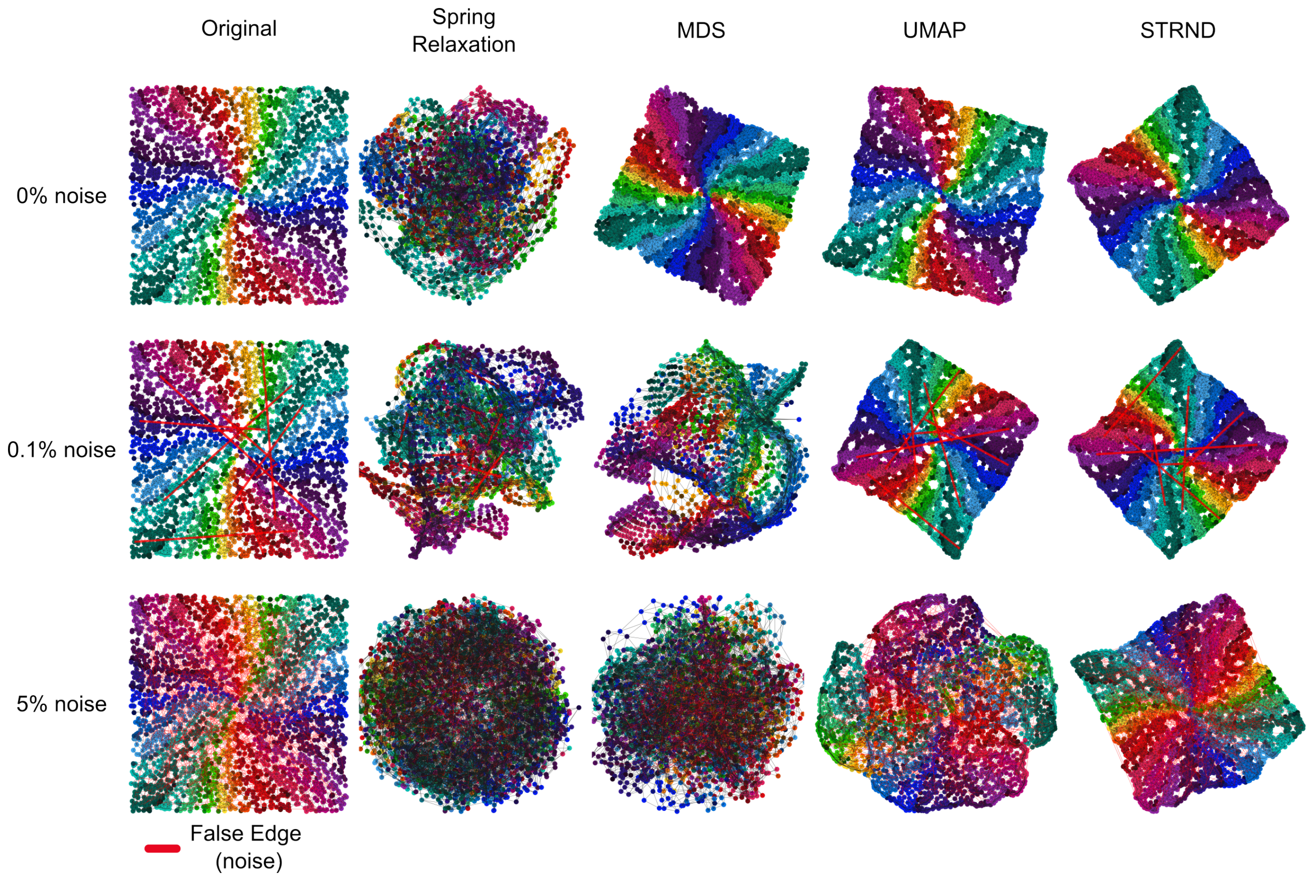

Figure S7: Image reconstruction visualizations for increasing noise levels (0%, 0.1%, 5%) on different image reconstruction methods.

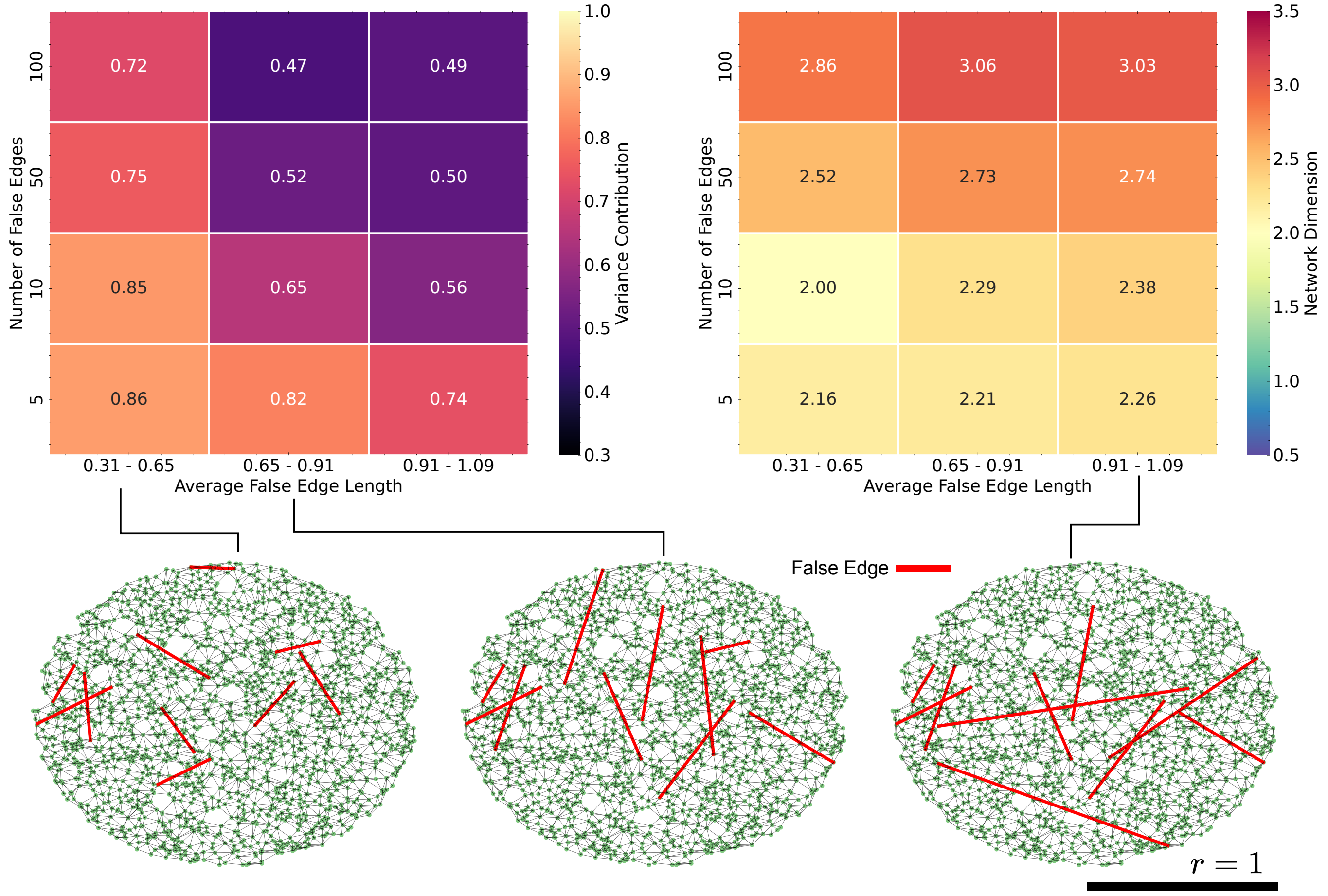

Figure S8: Impact of the number and length of false edges on spatial coherence measures. Left: variance contribution. Right: network dimension. A false edge length increase leads to a decrease in spatial coherence.

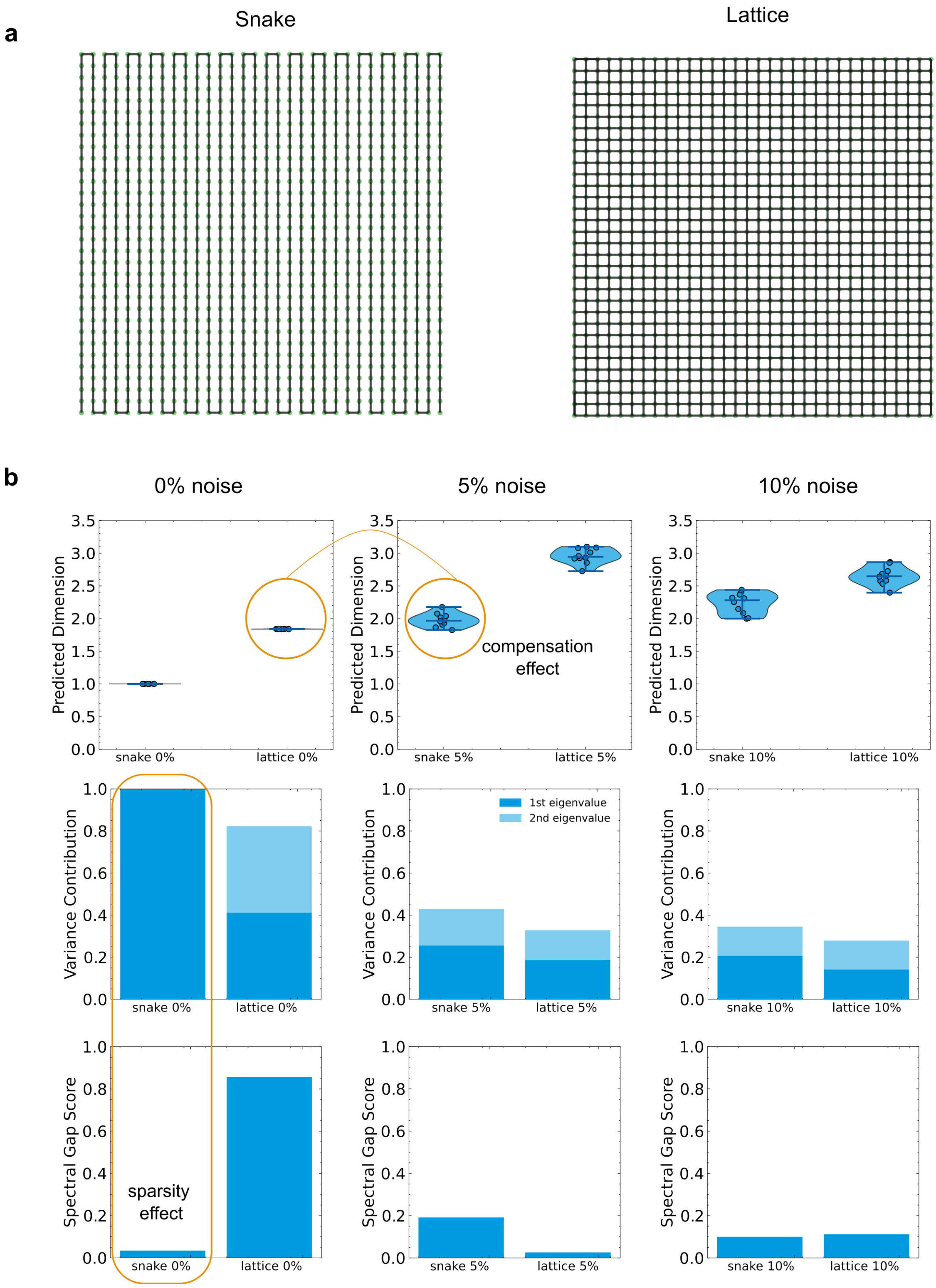

Figure S9: Compensation and sparsity effects in a toy example comparing snake-like lattice and square lattice topologies. **a** Node and edge distribution in physical space for both lattice types. **b** Predicted dimension under increasing noise levels. Moderate noise can make the one-dimensional snake lattice appear more two-dimensional. However, the spectral gap score remains low, indicating that the second and third eigenvalue are comparable, which is a consequence of the underlying one-dimensional ”chain” structure.

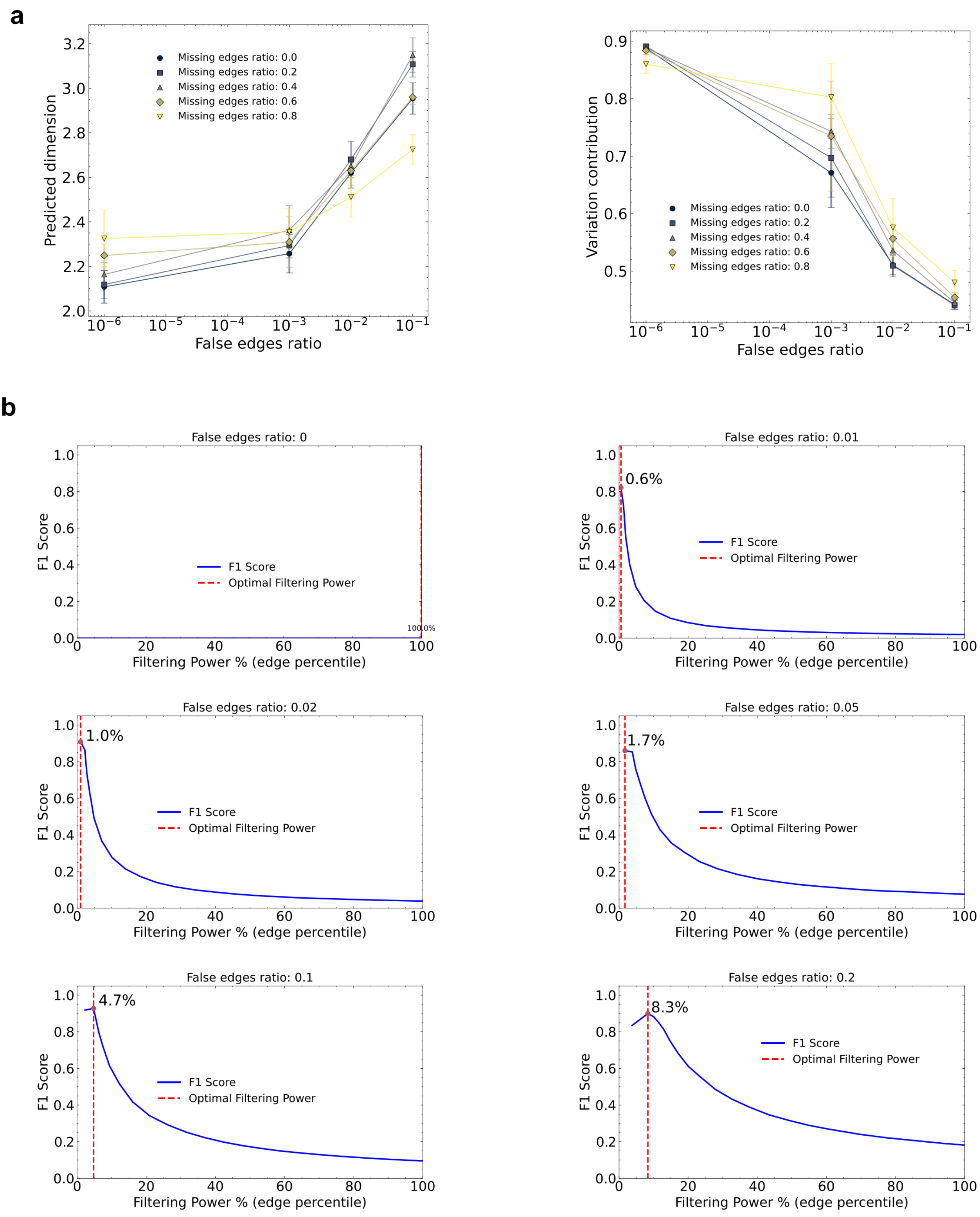

Figure S10: Supplement to the main figure panel Fig.3f. **a** Network dimension and Gram matrix variance contribution for graphs under different conditions of missing edges and false edges ratios. The experiment was repeated n=20 times for each data point to obtain a 95% confidence interval. **b** Quantitative evaluation of the optimal filtering power, according to the F1 score.

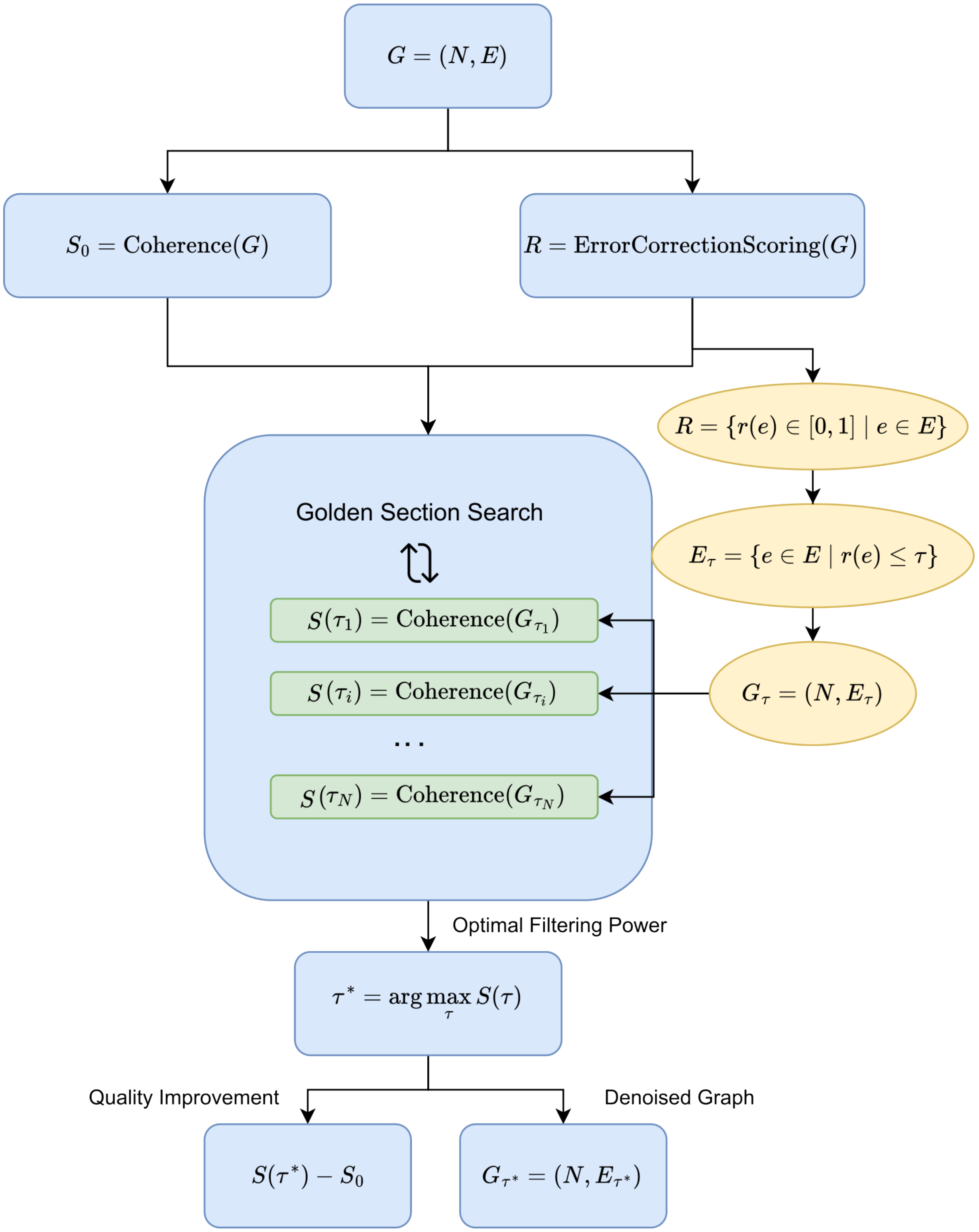

Figure S11: Filtering power optimization algorithm as a synergy between error correction strategies and spatial coherence metrics. Given a proximity graph $G = (N, E)$, a spatial coherence metric is computed S_0. Next, edge confidence scores are then assigned following an error correction strategy, producing a ranked list of edges R. A golden section search is used to identify the optimal filtering threshold τ^* that maximizes the spatial coherence of the filtered graph G_τ. The final output is the denoised graph G_{τ^*}, along with the improvement in spatial coherence $S(\tau^*) - S_0$.

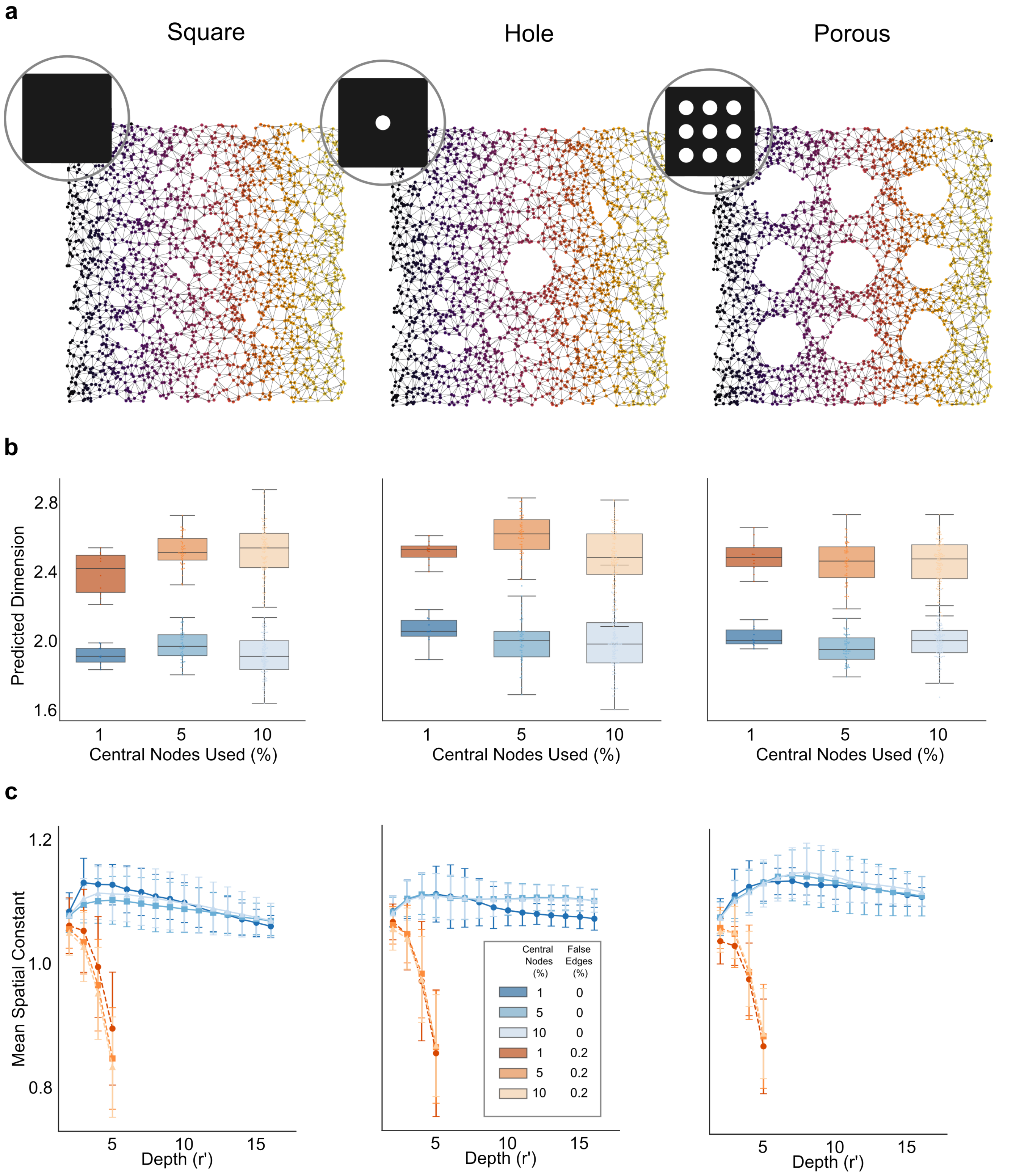

Figure S12: Effect of the number of central nodes on predicted dimension and spatial constant on usual and porous geometries. **a** Node and edge layouts in physical space for three geometries with varying porosity levels. **b** Predicted dimension for each geometry using different central node ratios (1%, 5%, and 10%) under two conditions: no noise and 0.2% noise. **c** Mean spatial constant under the same conditions and node ratios.
